# Guidelines for neuroprognostication in adults with traumatic spinal cord injury

**DOI:** 10.1007/s12028-023-01845-8

**Published:** 2023-11-13

**Authors:** Dea Mahanes, Susanne Muehlschlegel, Katja E. Wartenberg, Venkatakrishna Rajajee, Sheila A. Alexander, Katharina M. Busl, Claire J. Creutzfeldt, Gabriel V. Fontaine, Sara E. Hocker, David Y. Hwang, Keri S. Kim, Dominik Madzar, Shraddha Mainali, Juergen Meixensberger, Panayiotis N. Varelas, Christian Weimar, Thomas Westermaier, Oliver W. Sakowitz

**Affiliations:** 1 Departments of Neurology and Neurosurgery, UVA Health, University of Virginia, Charlottesville, VA USA; 2 Departments of Neurology, Anesthesiology and Surgery, University of Massachusetts Chan Medical School, Worcester, MA USA; 3 Department of Neurology, University of Leipzig, Leipzig, Germany; 4 Departments of Neurology and Neurosurgery, University of Michigan, Ann Arbor, MI USA; 5 School of Nursing, University of Pittsburgh, Pittsburgh, PA USA; 6 Departments of Neurology and Neurosurgery, College of Medicine, University of Florida, Gainesville, FL USA; 7 Department of Neurology, University of Washington, Seattle, WA USA; 8 Departments of Pharmacy and Neurosciences, Intermountain Health, Salt Lake City, UT USA; 9 Department of Neurology, Mayo Clinic, Rochester, MN USA; 10 Department of Neurology, University of North Carolina, Chapel Hill, NC USA; 11 Department of Pharmacy Practice, University of Illinois at Chicago, Chicago, IL USA; 12 Department of Neurology, University of Erlangen-Nuremberg, Erlangen, Germany; 13 Department of Neurology, Virginia Commonwealth University, Richmond, VA USA; 14 Department of Neurosurgery, University of Leipzig, Leipzig, Germany; 15 Department of Neurology, Albany Medical College, Albany, NY USA; 16 Institute of Medical Informatics, Biometry and Epidemiology, University Hospital Essen, Essen, Germany; 17 BDH-Clinic Elzach, Elzach, Germany; 18 Department of Neurosurgery, Helios Amper-Klinikum Dachau, Dachau, Germany; 19 Department of Neurosurgery, Neurosurgery Center Ludwigsburg-Heilbronn, Ludwigsburg, Germany

**Keywords:** Spinal cord injuries, Trauma, Prognosis, Functional status, Outcome

## Abstract

**Background:**

Traumatic spinal cord injury (tSCI) impacts patients and their families acutely and often for the long term. The ability of clinicians to share prognostic information about mortality and functional outcomes allows patients and their surrogates to engage in decision-making and plan for the future. These guidelines provide recommendations on the reliability of acute-phase clinical predictors to inform neuroprognostication and guide clinicians in counseling adult patients with tSCI or their surrogates.

**Methods:**

A narrative systematic review was completed using Grading of Recommendations Assessment, Development, and Evaluation methodology. Candidate predictors, including clinical variables and prediction models, were selected based on clinical relevance and presence of an appropriate body of evidence. The Population/Intervention/Comparator/Outcome/Timing/Setting question was framed as “When counseling patients or surrogates of critically ill patients with traumatic spinal cord injury, should < predictor, with time of assessment if appropriate > be considered a reliable predictor of < outcome, with time frame of assessment >?” Additional full-text screening criteria were used to exclude small and lower quality studies. Following construction of an evidence profile and summary of findings, recommendations were based on four Grading of Recommendations Assessment, Development, and Evaluation criteria: quality of evidence, balance of desirable and undesirable consequences, values and preferences, and resource use. Good practice recommendations addressed essential principles of neuroprognostication that could not be framed in the Population/Intervention/Comparator/Outcome/Timing/Setting format. Throughout the guideline development process, an individual living with tSCI provided perspective on patient-centered priorities.

**Results:**

Six candidate clinical variables and one prediction model were selected. Out of 11,132 articles screened, 369 met inclusion criteria for full-text review and 35 articles met eligibility criteria to guide recommendations. We recommend pathologic findings on magnetic resonance imaging, neurological level of injury, and severity of injury as moderately reliable predictors of American Spinal Cord Injury Impairment Scale improvement and the Dutch Clinical Prediction Rule as a moderately reliable prediction model of independent ambulation at 1 year after injury. No other reliable or moderately reliable predictors of mortality or functional outcome were identified. Good practice recommendations include considering the complete clinical condition as opposed to a single variable and communicating the challenges of likely functional deficits as well as potential for improvement and for long-term quality of life with SCI-related deficits to patients and surrogates.

**Conclusions:**

These guidelines provide recommendations about the reliability of acute-phase predictors of mortality, functional outcome, American Spinal Injury Association Impairment Scale grade conversion, and recovery of independent ambulation for consideration when counseling patients with tSCI or their surrogates and suggest broad principles of neuroprognostication in this context.

**Supplementary Information:**

The online version contains supplementary material available at 10.1007/s12028-023-01845-8.

## Introduction

According to the 2016 Global Burden of Disease study, the age-standardized incidence rate of traumatic spinal cord injury (tSCI) is estimated to be 13 cases per 100,000 people, or a total of 930,000 new cases worldwide. The global prevalence of tSCI in 2016 was approximately 27 million [1]. Average age at the time of injury has increased, primarily due to an increase in injuries among individuals aged 65 and older [2]. Deficits vary based on level of injury to the spinal cord. Based on data from the United States, incomplete tetraplegia is most common, impacting approximately 47% of patients, followed by complete and incomplete paraplegia [3]. The morbidity associated with tSCI is substantial and out of proportion to the incidence. Although traumatic brain injury (TBI) occurs approximately 30 times more often and the prevalence of patients with TBI sequelae is twice as high, tSCI is associated with more years lived with disability [1, 4].

Prognostication following tSCI focuses primarily on functional outcomes such as ambulation and the ability to perform activities of daily living. Short-term and long-term survival is also a consideration, especially for older, medically complex patients or patients with respiratory compromise. The ability to provide prognostic information may help patients and family with both short-term decision-making and long-term care planning. Accurate prognostication supports realistic goals and enables clinicians to better focus rehabilitation on either return of function or adaptation. In 1999, the Consortium for Spinal Cord Medicine published a comprehensive practice guideline focused on outcomes after spinal cord injury, with support from the Paralyzed Veterans of America [5].

Much of the research into outcomes after tSCI is drawn from observational data submitted to large registries or from centralized consortiums and networks. In the United States, the Spinal Cord Injury Model Systems (SCIMS) program includes centers that provide comprehensive care to individuals with SCI across the continuum of treatment [6]. The SCIMS program began in 1970 with a single center [6] and currently includes 14 institutions [7]. In addition to providing services to people with SCI and participating in research, each SCIMS center contributes data to a central database. The SCIMS database is managed through the National Spinal Cord Injury Statistical Center and is available to researchers [7]. The North American Clinical Trials Network, a network of ten US and Canadian centers, was established in 2005 to promote advancement in tSCI management through research. Now affiliated with the Christopher and Dana Reeve Foundation, the North American Clinical Trials Network maintains an SCI registry focused on the natural history of recovery in the context of current treatments, information that can then be used for comparison in future interventional studies [8]. In Canada, more than 30 centers contribute information to the Rick Hansen Spinal Cord Injury Registry (RHSCIR) using a prospectively established standardized data set. Although initially focused on tSCI, the RHSCIR has expanded to include nontraumatic SCI. Similar to many other consortiums or networks, centers that belong to the RHSCIR commit to the implementation of best practices for SCI care [9]. Outside of North America, the European Multicenter Study about Spinal Cord Injury (EMSCI) was founded in 2004 as a collaborative effort between several leading spinal cord injury centers across Europe. The initial aim of the organization was to establish a comprehensive database of clinical and demographic information on patients with SCI to improve the quality of care for these individuals. Since its inception, EMSCI has grown to include more than 20 member centers in countries including Austria, Belgium, Denmark, France, Germany, Italy, the Netherlands, Norway, Spain, Sweden, Switzerland, and the United Kingdom. The organization has also expanded its research focus beyond data collection to include the design and implementation of clinical trials and observational studies aimed at improving the treatment and outcomes of tSCI. Over the years, EMSCI has been instrumental in promoting collaboration and knowledge sharing among researchers and health care professionals across Europe and beyond [10].

### Scope, Purpose, and Target Audience

The scope of these Grading of Recommendations Assessment, Development and Evaluation (GRADE) guidelines is the prognostication of neurological outcome in critically ill adult patients with tSCI. The purpose of these guidelines is to provide evidence-based recommendations on the reliability of predictors of neurological outcome in critically ill adult patients with tSCI, to aid clinicians in formulating a prognosis. The target audience consists of clinicians responsible for such counseling.

### How to Use These Guidelines

These guidelines provide recommendations on the reliability of select demographic and clinical variables as well as prediction models when counseling patients, families, and surrogates of individuals with tSCI. We categorized these predictors as reliable, moderately reliable, or not reliable. We based this categorization on a GRADE-based assessment of certainty in the body of evidence, as well as effect size (quantification of predictor accuracy) across published studies, as detailed in Supplementary Appendix 1 and Table 1. Reliable predictors and prediction models, for the purposes of these guidelines, may be used to formulate a prognosis when the appropriate clinical context is present in the absence of potential confounders. These are predictors with clear, actionable thresholds or clinical/radiographic definitions and a low rate of error in prediction of outcomes, with at least moderate certainty in the body of evidence. When prognosis is formulated on the basis of one or more reliable predictors, the clinician may describe the outcome as “very likely” during counseling. Given the inherent limitations in neuroprognostication research, the clinician must nevertheless acknowledge the presence of uncertainty—even if low—in the prognosis during counseling. Moderately reliable individual predictors may be used for prognostication *only* when additional reliable or moderately reliable individual predictors are present, in addition to the appropriate clinical context. These are also predictors with clear, actionable thresholds or clinical/radiographic definitions and a low rate of error in prediction of outcomes, but with lower certainty in the body of evidence, frequently as a result of smaller studies that result in imprecision or other risk of bias, often rooted in methodology. When the prognosis is formulated on the basis of multiple moderately reliable predictors, the clinician may describe the outcome as “likely” during counseling but must acknowledge “substantial” uncertainty in the prognosis. Moderately reliable clinical prediction models that generate predicted probabilities of outcomes, in contrast, may be used for prognostication during counseling of patients, families, and surrogates of individuals with tSCI in the absence of other reliable or moderately reliable predictors. However, it is recommended that the clinician describe the predicted probability of the outcome as “an objective estimate only, subject to considerable uncertainty.” Although the panelists recognize that those predictors that do not meet the criteria to be described as reliable or moderately reliable are often used by clinicians in formulating their subjective impressions of prognosis, they have nevertheless been deemed not reliable for the purposes of these guidelines and cannot be formally recommended for prognostication on their own. Variables deemed not reliable, however, may be a component of reliable or moderately reliable prediction models.

**Table 1:** Predictor Characterization and Use—Reliable and Moderately Reliable Predictors

Category of predictor/ model	GRADE criteria					Point estimates of accuracy in the body of evidence	Use during counseling of patients or surrogates?	Presence of additional specific reliable or moderately predictors required for use during counseling	Suggested language during counseling of patients or surrogates	
	Risk of Bias	Inconsistency	Imprecision	Indirectness	Quality of Evidence- Overall				Likelihood of outcome	Disclaimer of Uncertainty during counseling
Reliable	One downgrade permitted	Downgrade NOT permitted	Downgrade NOT permitted	Downgrade NOT permitted	Moderate or High	High. Prediction models require AUC > 0.8, no evidence of miscalibration in external validation studies	Yes	Preferred, but not absolutely required	“Very likely” or for clinical prediction models use predicted probability of outcome	Present, but low
Moderately reliable individual predictors	One downgrade permitted	Downgrade NOT permitted	One downgrade permitted	One downgrade permitted	Any	High	Yes	Yes	“Likely”	Substantial
Moderately reliable clinical prediction models	One downgrade permitted	Downgrade NOT permitted	One downgrade permitted	One downgrade permitted	Any	High. Prediction models require AUC > 0.7, some miscalibration allowed in external validation studies	Yes	No	Use predicted probability of outcome	“The predicted probability is an objective estimate, subject to considerable uncertainty”
Not reliable	Downgrade permitted	Downgrade permitted	Downgrade permitted	Downgrade permitted	Any	Any	No^a^	Not applicable	Not applicable	Not applicable

*AUC*, area under the curve, *GRADE*, Grading of recommendations assessment, development, and evaluation

^a^Many predictors designated “not reliable” are practically used by clinicians in formulating and communicating real-world subjective impressions of prognosis. The purpose of these guidelines is to identify predictors, if any, that meet reliable or moderately reliable criteria

## Methods

An in-depth description of the methodology used in these guidelines is available in Supplementary Appendix 1.

### Selection of Guideline Questions

Candidate predictors were selected based on clinical relevance *and* the presence of an appropriate body of literature. Candidate predictors and prediction models were considered “clinically relevant” if the predictor or components of the model were (1) accessible to clinicians, although universal availability was not required, *and* (2) likely to be considered by clinicians when formulating a neurological prognosis for critically ill adult patients with tSCI. An appropriate body of literature was considered present for any clinical variable that was evaluated in at least two studies that included a minimum of 50 study participants and established as an independent predictor in a multivariate analysis that incorporated age and measures of injury severity (level and/or completeness of injury). For clinical prediction models, an appropriate body of literature was considered present for models with at least one external validation study of at least 50 patients in addition to the initial report on development of the model (also with a minimum of 50 patients). Because of the relatively low incidence of tSCI, studies of at least 50 study participants were included to broaden the available literature. The panel’s goal was to delineate factors for prognosis of the natural course following tSCI based on acute-phase assessments in the critical care environment. Treatment of patients with trauma is heterogeneous, but the impact of therapeutic interventions on outcome was outside the scope of these prognostication guidelines. Based on these criteria, the following candidate predictors were selected.

#### Clinical Variables and Description

1. Age at time of injury. Age at the time of injury may be used as a continuous variable or, more often, divided into categories. These categories vary widely across studies and may be impacted by changes in the epidemiology of SCI, population characteristics, and evolving societal perspectives on aging. Although age is often dichotomized at 65 years, other thresholds, such as 60 or even 50 years, have been suggested.
2. Comorbidities. Comorbid conditions such as cardiac, pulmonary, or renal disease may be present at the time of tSCI. These conditions may complicate management and potentially impact mortality. Comorbidities at the time of acute hospitalization are often reported in large-scale registries or can be extracted from the electronic health record. Common methods for measuring this predictor include use of the Charlson Comorbidity Index (CCI) (Supplementary Table 1) or a count of the number of comorbid conditions reported.
3. Concomitant injury. The presence of concomitant brain injury or other multisystem trauma may impact outcomes. Concomitant TBI is typically classified by its presence, the Glasgow Coma Scale (GCS) score following initial resuscitation, or by the three traditional gradations of severity: mild (GCS 13–15), moderate (GCS 9–12), and severe (GCS 3–8). Injury scoring systems such as the Abbreviated Injury Scale and the Injury Severity Score (ISS) provide information about the severity of trauma and body regions impacted. The Abbreviated Injury Scale, developed in the 1960s in response to an increase in automobile accidents, assigns an injury severity score by body region (head/neck, face, chest, abdomen/pelvic organs, extremities/pelvic girdle, and external). Each region is scored from 0 to 6 according to injury severity (0 none, 1 minor, 2 moderate, 3 serious, 4 severe, 5 critical, and 6 maximal/untreatable). The ISS, used by trauma systems internationally, is the sum of the squares of Abbreviated Injury Scale scores for the three body regions with the highest severity scores. The resulting sum score for injured patients ranges from 1 to 75 [11, 12]. For the purposes of these guidelines, we did not limit our search to a specific definition of concomitant injury to avoid excluding potentially relevant predictors.
4. Neurological level of injury (NLI). NLI reflects the most caudal segment of the spine with normal sensation and at least antigravity motor strength, provided that rostral movement and sensation is normal. The International Standards for Neurological Classification of Spinal Cord Injury (ISNCSCI) scale, commonly called the “American Spinal Injury Association (ASIA) Scale,” provides a structured approach to assessment and determination of both NLI and severity (completeness) of injury [13]. The ISNCSCI scale may be impacted by spinal shock and requires training to perform accurately, so it is not reported in all studies of early prognostication after SCI. Some studies identify the specific NLI by spinal segment, whereas others categorize injury by region (cervical, thoracic, lumbar, thoracolumbar, conus, or other). The broadest differentiation based on level of injury is tetraplegia (cervical and cervicothoracic SCI) or paraplegia (thoracic or thoracolumbar SCI). Patients with complete injuries may show signs of partial sensory or motor innervation below the NLI, described as zones of partial preservation (ZPP). It has been suggested that occurrence and segmental width of ZPPs may predict future functional improvements. Timing of assessment is of critical importance for this predictor, but significant variability exists in the literature. For the purposes of prognostication, determination of NLI should be deferred for 72 h. The initial examination may be impacted by spinal shock and by other factors such as concomitant injuries, medications, and ongoing resuscitation. An examination performed at 72 h after injury is likely more predictive of long-term functional outcomes than earlier assessments [14–16]. Previous guidelines for prediction of outcomes after tSCI recommend comprehensive assessment 3 to 7 days after injury [5]. One caveat is that for the purpose of scientific analysis, assessments performed at later time intervals are more likely to reflect treatment effects than assessments done at the time of initial presentation. For the purposes of this systematic review, the NLI was defined as the first reported ISNCSCI scale or a similar scoring system. In most cases, the first NLI was recorded at admission or within 72 h, although this information was not reported in all studies. Studies were not excluded if the time of determination of NLI was not specified, provided the NLI was determined during the initial postinjury hospitalization.
5. Magnetic resonance imaging (MRI) findings. Radiological evaluation by computed tomography is standard in trauma systems. MRI diagnostics have evolved as state-of-the-art tools in the management of tSCI but may be limited outside of core working hours in some areas. Costs, staffing issues, safety concerns, and sometimes logistics of patient care may be prohibitive. However, MRI provides good soft tissue contrast and the ability to evaluate details such as neurovascular injury, ongoing compression, extent of secondary injuries, and resolution of injury (signs of cord compression, lesion size, edema) following definitive treatment. MRI findings with a potential role in prognostication include spinal canal compromise, intramedullary signal change (e.g., hemorrhage, ischemia), maximum spinal cord compression, and extension of edema. MRI protocols differ between hospital systems and the specific protocol used needs to be carefully considered in prognostic studies. Advanced microstructural, biochemical, or functional imaging techniques of the spinal cord such as magnetic resonance spectroscopy, diffusion tensor imaging, positron-emission of single-photon-emission tomography, or functional MRI are in development. For the purposes of these guidelines, specific MRI findings were not defined prior to the literature search to avoid excluding potentially relevant predictors.
6. Severity of injury to the spinal cord (complete or incomplete). Following tSCI, loss of motor and sensory function may be complete or incomplete. Incomplete injury indicates conduction of electrical impulses past the area of cord injury. Completeness of injury is most often reported using the ASIA Impairment Scale (AIS) score (Supplementary Table 2), although earlier research may report the Frankel grade, a predecessor to AIS grading. A patient with a complete injury (AIS A) has no sensation or movement below the lesion. Incomplete injury reflects varying degrees of sensory or motor preservation and must include evidence of preserved S4-5 function, which can be motor (voluntary anal contraction), sensory (light touch, pin prick sensation, or deep anal pressure sensation), or both. Preservation of sensation with complete loss of motor function below the NLI is categorized as AIS B, sensory incomplete. The classifications AIS C and AIS D denote motor incomplete injuries. If less than half of key muscle groups below the NLI have at least antigravity strength, the injury is classified as AIS C; if at least half of the key muscles below the NLI have at least antigravity strength, the injury is classified as AIS D. A grade of AIS E indicates resolution of SCI-related deficits [13]. Antigravity muscle strength (muscle function grade of 3 on a 5-point scale) is the minimum necessary for recovery of functional activities. Similar to NLI, the timing of assessment is important. For the purposes of prognostication, determination of completeness of injury (AIS grade) should be deferred for 72 h, with the previously noted considerations and limitations in the available literature.

Note on early surgical decompression as a predictor of outcome: Treatment of tSCI has been driven by medical management, surgical restoration of spinal column stability, and surgical decompression of the spinal canal to counter existing or developing pressure on the spinal cord. Although the benefit of early surgical decompression has been a topic of debate, there is consensus that it is safe and may improve neurological outcomes [17]. The impact of therapeutic interventions was outside the scope of these guidelines. However, clinicians should be aware of the potential beneficial impact of treatment approaches, specifically early surgical decompression, on neurological outcomes. Information about treatment approaches should be incorporated into research into prognosis after tSCI and should include measurement of level and completeness of injury before and after significant treatment interventions.

#### Clinical Prediction Models

The Dutch Clinical Prediction Rule (DCPR), derived from the EMSCI data set, was introduced in 2011 to aid in early prediction of independent ambulation among adult patients after tSCI. Variables in the final model were age dichotomized at 65 years, motor scores of the quadriceps femoris (L3) and gastrocnemius (S1) muscles, and light touch sensation in the corresponding dermatomes [18]. Neurological assessment was performed within 15 days of injury. International (external) validation studies have been conducted in North America (USA and Canada) and Australia [19–22]. The DCPR generates a total score of − 10 to 40 that can be used to predict probability of ambulation at 1 year. Table 2 provides an overview and examples of DCPR score calculation, and Table 3 indicates the predicted probability of independent ambulation at 1 year based on selected DCPR scores.

**Table 2:** Dutch clinical prediction rule (DCPR) variables and scoring

Variable	Range of test scores	Weighted coefficients	Minimum score	Maximum score
Age > 65 years	0–1	− 10	− 10	0
Motor score L3	0–5	2	0	10
Motor score S1	0–5	2	0	10
Light touch score L3	0–2	5	0	10
Light touch score S1	0–2	5	0	10
Total		− 10	40	
van Middendorp JJ, Hosman AJF, Donders ART, Pouw MH, Ditunno JF, Curt A, et al. A clinical prediction rule for ambulation outcomes after traumatic spinal cord injury: a longitudinal cohort study. Lancet. 2011;377:1004–10				

Scoring of motor and sensory scores is in accordance with the international standards for neurological classification of spinal cord injuries (ISNCSCI), from the American Spine Injury Association (ASIA) and the International Spinal Cord Society (ISCoS). Assessment is performed within 15 days following injury. The best motor and sensory score (left vs. right) is used

The motor score is calculated as follows: 0 = Total paralysis; 1 = Palpable or visible contraction; 2 = Active movement with gravity eliminated; 3 = Active movement against gravity; 4 = Active movement against some resistance; 5 = Active movement against full resistance; NT = Not testable. Motor function is tested with knee extension at L3 and plantar flexion at S1

The sensory score is calculated as follows: 0 = Absent; 1 = Altered; 2 = Normal; NT = Not Testable. Sensation is typically tested at the medial femoral condyle for the L3 dermatome and the lateral aspect of the heel for the S1 dermatome

The age, motor L3, motor S1, light touch L3 and light touch S1 scores are multiplied by the respective weight coefficients. The sum of these numbers is then the final score, which ranges from − 10 (patient 65 years or older with complete paralysis and absent sensation at both L3 and S1) to 40 (patient younger than 65 years with active movement against full resistance and normal sensation at both L3 and S1)

For example, a 65-year-old patient (A) with a 1 out of 5 strength in knee extension (L3) and 0 out of 5 strength in plantarflexion (S1) and scant sensation in both dermatomes will obtain a score of 2 (− 10 + 2 + 0 + 5 + 5), while a 50-year-old patient (B) with 2 out of 5 strength in L3 and 2 out of 5 strength in S1 and unimpaired sensation in the corresponding dermatomes will obtain a score of 28 (0 + 4 + 4 + 10 + 10)

**Table 3:** Dutch Clinical Prediction Rule (DCPR): predicted probability of ambulation by selected scores

DCPR Score	Predicted probability of independent ambulation at 1 year (%)
− 10	< 1
− 5	1
0	4
2	6
5	13
10	35
15	68
20	89
25	97
28	99
30	> 99
35	100
40	100

Predicted probability of independent ambulation corresponding to various DCPR scores. See Table 2 for method of calculation of DCPR score. Predicted probabilities are calculated based on the following model

Predicted probability = EXP(− 3.273 + 0.267 × DCPR − score)/(1 + EXP[− 3.279 + 0.267 × DCPR − score])

Point estimates for example cases A and B from Table 2 are given

van Middendorp JJ, Hosman AJ, Donders AR, Pouw MH, Ditunno JF, Jr., Curt A, et al. A clinical prediction rule for ambulation outcomes after traumatic spinal cord injury: a longitudinal cohort study. Lancet. 2011;377(9770):1004–10

#### Guideline Questions

The Population/Intervention/Comparator/Outcome/Time frame/Setting question was framed for the specific candidate predictors as follows:

When counseling patients, family members, and/or surrogates of adults with acute traumatic spinal cord injury, should <*predictor, with time of assessment if appropriate*> be considered a reliable predictor of <*outcome, with time frame of assessment*>?

### Selection of Outcomes

Outcomes were selected using the GRADE 1–9 scale with input from experts on the writing panel and the patient representative. The outcomes rated “critical” were mortality at discharge from the acute care hospital or later (average rating 7.67), functional outcome at discharge from rehabilitation or later (average rating 8.67), improvement in ASIA Impairment Scale (AIS grade conversion) at discharge from rehabilitation or later (average rating 7.0), independent ambulation at discharge from rehabilitation or later (average rating 8.0), and bowel and bladder control (average rating 8.0). Outcomes were defined as follows:

- Mortality. Because the factors impacting mortality related to tSCI vary based on time from injury, the reliability of predictors of acute in-hospital mortality (occurring during the initial acute hospitalization after injury) has been described separately from predictors of long-term mortality (cumulative mortality measured at any time point after hospital discharge).
- Functional outcome. Assessment of functional outcome after tSCI is most often performed with the Functional Independence Measure (FIM) [23–25] or Spinal Cord Independence Measure (SCIM) [26] (Supplementary Tables 3 and 4). Both FIM and SCIM are validated tools that measure the patient’s ability to perform tasks important to daily living. The FIM can be used with a wide range of patients and is divided into a motor subscale (self-care, sphincter control, transfers, and locomotion) and a cognition subscale (communication and social cognition). Scores range from 1 (totally dependent) to 7 (device- and helper-independent). The FIM motor score, constructed from 13 subscales, therefore ranges from 13 to 91. Functional independence is assumed once scores of 6 or higher are reached on all subscales. The SCIM assesses three domains: self-care, respiration and sphincter management, and mobility. Because FIM and SCIM scores are expected to vary based on NLI, no specific score was used to define good versus poor outcomes. Scores are expected to improve in the first 3 months and plateau within 6–9 months with continued rehabilitation. In clinical studies, functional outcome assessments based on the FIM score are typically performed at 1 year. When 1-year outcomes are unavailable, 6 month-scores are acceptable [18, 27].
- AIS grade conversion. Studies reporting only change in motor score on the ISNCSCI scale or its predecessors were excluded because an isolated change in motor score is difficult to interpret and may not reflect improved function. A number of studies reported AIS grade conversion as a surrogate marker of improved function. These studies were included because AIS grade provides broad categories of function for discussion with patients and families. In addition, improvement in AIS grade may reflect increased potential for future participation in self-care. As with other functional outcome measures (see above), most improvement is seen within 3 months following injury with a plateau 6–9 months following injury. This measure is widely used clinically during acute care and at follow-up.
- Independent ambulation. Independent ambulation was broadly defined as the ability to walk for short distances with or without assistive devices. Most studies report ambulation using a subscale of either the FIM or SCIM, with scores dichotomized into two groups (independent ambulation/walkers or no independent ambulation/nonwalkers) for analysis. The specific definition of independent ambulation varies slightly by the scale used (FIM or SCIM) and the version used (SCIM I, II, or III). Studies that utilize the FIM typically define independent ambulation as a locomotion (walking) score of 6 or 7, representing modified or complete independence [21, 22]. Alternatively, independent ambulation has been defined by a locomotion-walking score of 5 (supervision only) or higher with at least 50 m of unassisted walking [28]. All versions of SCIM include multiple subscales for mobility. Independent ambulation is typically defined as an indoor mobility score > 3, reflecting the ability to walk short distances inside without supervision, with or without assistance devices such as a walking frame, crutches, or canes [18].
- Bowel and bladder control. Bowel and bladder control, broadly defined as the ability to maintain continence with or without the use of adjuncts, was included as an outcome of interest because continence is a functional outcome that impacts quality of life [29, 30]. Although the initial literature search returned several articles addressing this outcome, none of the studies met criteria for inclusion, either due to methodological flaws or because they did not address prognostication based on information available during critical care management.

### Systematic Review Methodology

**Fig. 1:**
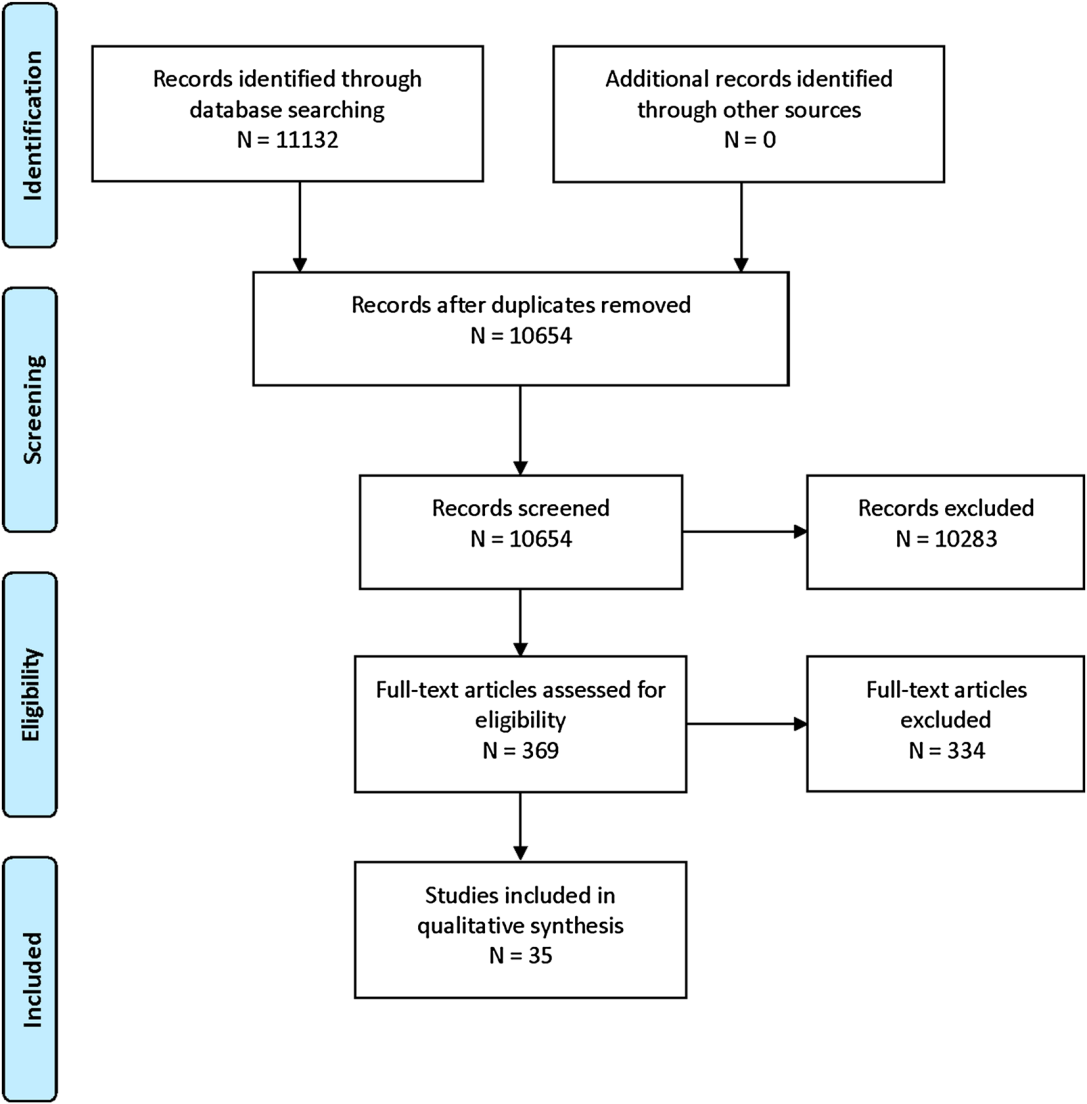
PRISMA flow diagram—guidelines for neuroprognostication: traumatic spinal cord injury

An in-depth description of systematic review methodology for these guidelines is in Supplementary Appendix 1. The librarian search string used for this systematic review is in Supplementary Appendix 2 and the PRISMA flow diagram is in Fig. 1. Full-text screening was performed with the following exclusion criteria: sample size less than 50, focuses on a highly selected subgroup (such as penetrating trauma or central cord syndrome in patients with underlying degenerative cervical myelopathy), studies of predictors not established as independent with multivariate analysis, studies focused on a genetic polymorphism as a predictor, and studies of clinical prediction models that did not report model discrimination. Studies of laboratory biomarkers were included only if the biomarker was considered clinically relevant and had been evaluated in more than one published study that met other criteria. Spinal cord injury unrelated to trauma (for example, ischemic injury) was excluded.

A summary of individual studies of predictors is in Supplementary Appendix 3. The GRADE evidence profile and summary of findings table is in Table 4.

**Table 4:** Evidence profile/summary of findings

Outcome	Predictor or model	Quality of evidence					Summary of findings (narrative of effect size)
		RoB	Inconsist-ency	Indirect-ness	Impreci-sion	QoE- Summary	
Mortality, acute in-hospital	Age	↓	–	–	↓	Low	↑ with age, but ≥ 75% survive to discharge in most studies even among older patients
	Comorbidities	↓	↓	–	–	Low	↑ with 2 or ≥ 3 vs. no comorbidities (HR 1.73–2.19; OR 2.2–2.7), ≥ 75% survive to discharge. Inconsistent findings in smaller studies
	Concomitant Injury	↓	–	–	↓	Low	↑ with multisystem injury (HR 1.4–1.85), higher Injury Severity Score and TBI. Overall survival > 80%
	NLI	↓	↓	–	↓	Very low	↑ with high vs. low cervical injury (OR 2.14–5.24), Results vary for other levels
	Severity of SCI	↓	–	–	↓	Low	↑ with complete injury, especially cervical, but > 75% survive even with complete cervical injury
Mortality, long-term	Age	↓	–	–	↓	Low	↑ with age at time of injury, but age categories vary
	Comorbidities	↓	↓	–	–	Low	Variable results and limited data
	Concomitant Injury	↓	–	–	–	Moderate	No significant ↑ in long-term mortality with concomitant injury
	NLI	↓	↓	–	↓	Very low	Variable results. ↑ for C1–4 vs. other levels in largest study (HR 1.6), survival in C1–4 group ~ 70%
	Severity of SCI	↓	–	–	↓	Low	Study size/methodology varies. HR of 0.3–0.6 for incomplete vs. complete injury in largest study, with overall mortality of 16.2%
Functional outcome, score-based	Age	↓	–	–	–	Moderate	↓ motor recovery (FIM motor score) with age ≥ 65 years. AIS grades B/C patients at younger age fared better
	NLI	↓	–	–	↓	Low	↑ future motor outcome for thoracolumbar injuries vs. cervical. Substratified by neurological structures assessed by MRI, cauda equina > conus medullaris > thoracic myelon
	Severity of SCI	↓	–	–	–	Moderate	↑ future motor outcome with ↓ injury severity (AIS)
Improvement in AIS	Age	↓	–	–	–	Moderate	Improvement in AIS up to 12–24 months is independent of age at time of injury and age*gender interaction
	MRI	↓	–	–	↓	Low	↑ in AIS less likely with signatures of tSCI such as maximum spinal cord compression (> 50%) or extension of spinal cord edema
	NLI	↓	–	–	–	Moderate	↑ in AIS at 6–12 months is 1.5–fourfold more likely with tSCI to conus level or below compared with cervical or thoracic injuries
	Severity of SCI	↓	–	–	↓	Low	↑ in AIS is 4–eightfold more likely with AIS B–D compared with AIS A
Independent ambulation	MODEL: Dutch Clinical Prediction Rule (DCPR)	↓	–	–	–	Moderate	DCPR allows for accurate early prediction of an individual patient's ability to walk at 1 year post injury (AUC > 0.95); simplifying the rule by leaving out age or one motor score and one sensory score may yield similar results (AUC 0.87–0.94); prediction accuracy may vary by initial injury severity (AIS A + D > B + C)

Long-term mortality reflects cumulative mortality measured at any time point following hospital discharge

*AIS*, American Spinal Injury Association Impairment Scale, *AUC*, area under the curve, *DCPR*, Dutch clinical prediction rule, FIM, Functional independence measure, *HR*, hazard ratio, *MRI*, magnetic resonance imaging, *NLI*, neurological level of injury, *OR*, odds ratio, *QoE*, quality of evidence, *RoB*, risk of bias, *TBI*, traumatic brain injury, *tSCI*, traumatic spinal cord injury

### Evidence to Recommendation Criteria

1. Quality of evidence/certainty in the evidence and effect size: For the purpose of these guidelines, predictors described as “reliable” have both a higher overall certainty in the evidence and greater effect size than “moderately reliable” predictors (Table 1). For “reliable” predictors and prediction models, one downgrade was permitted for risk of bias, but none for inconsistency, imprecision or indirectness, and the overall quality of evidence had to be high or moderate. “Reliable” prediction models were required to demonstrate an area under the receiver operating curve (AUC) of > 0.8, and no evidence of miscalibration in external validation studies that reported calibration. Single downgrades within each of the domains of risk of bias, imprecision, and indirectness were permitted for “moderately reliable” predictors, but a downgrade for inconsistency was not. In addition, “moderately reliable” prediction models were required to demonstrate an AUC > 0.7, and some miscalibration in some external populations was allowed, given the lower risk of withdrawal of life support in this disease. Predictors that did not fit “reliable” or “moderately reliable” criteria were classified as “not reliable.”
2. Balance of desirable and undesirable consequences: Accurate prognostication of functional outcomes after acute tSCI supports the ability of patients, families, and clinicians to plan for future needs and to better focus rehabilitation activities on recovery of function versus adaptation. Inaccurate prediction of a poor functional outcome may lead to psychological consequences (depression, hopelessness) that negatively impact engagement in rehabilitation. Among patients requiring early ventilator support, inaccurate prediction of a poor functional outcome could become a self-fulfilling prophecy, with mortality due to withdrawal of life-sustaining treatment. Although the patient’s ability to engage in self-determination is a desirable outcome, early withdrawal of life-sustaining treatment may be an undesirable consequence when based on imperfect prognostic information. The panel and patient representative considered early withdrawal of life-sustaining treatments in patients with tSCI to be less likely than other populations, such as those with severe TBI or comatose cardiac arrest survivors, but still relevant for consideration. Although clinical variables may be associated with higher in-hospital and long-term mortality, the panel exercised caution when making recommendations about the use of these variables as predictors of mortality due to concerns about early withdrawal of life-sustaining treatment in patients with the potential for recovery or an acceptable quality of life with SCI-related disability.
3. Values and preferences: The panel and patient representative noted that many patients with isolated tSCI are conscious and able to participate in discussion with providers. They agreed that most individuals with tSCI, along with their families and surrogates, would likely value information about predicted functional outcomes even when that information is incomplete or uncertain because it supports planning for the future. The patient representative stressed the importance of providing balanced information when discussing prognosis, specifically addressing both the potential for a good quality of life and the burdens associated with functional losses.
4. Resource use: The predictors and models that have been tested in prognostication of spinal cord injury reflect information already collected as part of routine care. Clinical neurological assessment is required for care and therefore requires no additional expenditure. Although MRI has cost associated, it is considered a basic assessment tool for tSCI in most centers. Conversely, the costs associated with the long-term sequelae of SCI are substantial and having information about prognosis can assist with planning [3].

### Good Practice Statements

In accordance with recommendations of the GRADE network these statements were considered by the panel to be actionable, supported by indirect evidence where appropriate, and essential to guide the practice of neuroprognostication. The good clinical practices reflected in these statements lacked a meaningful body of direct supporting evidence—typically because of insufficient clinical equipoise—but were considered by the panel to be unequivocally beneficial [31].

Good practice statement #1: We recommend that prognostication should be performed with consideration of the complete clinical condition and never based on a single variable (strong recommendation, evidence cannot be graded).

- Rationale: Neuroprognostication in tSCI is complicated by heterogeneity in injury patterns, management strategies, and study methodology. Clinicians must therefore use caution when formulating a prognosis with the predictors addressed in these guidelines. In addition, the overall body of literature on tSCI outcomes is poor quality. Most studies performed retrospective analysis of registry databases that, even with prospective enrollment, may exclude important variables. Studies based on retrospective chart review have additional limitations, especially for determination of outcomes. Given these limitations, prognostication can be improved through consideration of multiple predictors. Decisions should always be made with attention to individual patient characteristics, as well as preferences and values.

Good practice statement #2: We suggest that when discussing prognosis with patients and surrogates in the immediate postinjury period, clinicians provide information about the significant challenges of likely functional deficits as well as the potential for improvement and for long-term quality of life with SCI-related deficits (strong recommendation, evidence cannot be graded).

- Rationale: Good communication is essential when conveying information about the short-term and long-term impacts of tSCI. Research specific to tSCI is limited, although two small studies emphasize hearing initial information from a physician and communication that is truthful but supports hope [32, 33]. Unlike some neurological diagnoses that impact cognitive function, the patient with tSCI is often able to actively participate in discussion about prognosis. Because the impact of tSCI on family members and other caregivers is substantial, they should be included in communication with the patient’s consent, when appropriate. While clinical considerations are often the initial focus, the financial impact of tSCI is significant and may include loss of income as well as the costs for equipment and caregivers. By devoting time to ongoing discussion and providing information about the range of possible functional outcomes, clinicians can best support patients with tSCI and their families as they plan for the future. The patient’s NLI and AIS Grade provide information about anticipated functional deficits, although uncertainty must be acknowledged. Early involvement of rehabilitation professionals may be helpful in discussion of the long-term impacts of tSCI.

## Recommendations: Clinical Variables as Predictors

### Outcome: Mortality (Acute, in-Hospital)

Question: When counseling patients, family members, and/or surrogates of adults with acute tSCI, should older age at the time of injury alone be considered a reliable predictor of acute in-hospital mortality?

- Recommendation: When counseling patients, family members, and/or surrogates of adults with acute tSCI, we suggest that older age alone not be considered a reliable predictor of acute in-hospital mortality (weak recommendation; low quality evidence).
- Rationale: The body of evidence was downgraded for risk of bias, with various studies demonstrating potential bias in the Quality in Prognostic Studies (QUIPS) domains of study participation, prognostic factor measurement, confounding, statistical analysis and reporting, and self-fulfilling prophecy. Many studies had high risk of bias in the domain of confounding because treatment effect was not considered, and in statistical analysis and reporting because the studies were underpowered, or the analysis was not well described. Most studies also had moderate to high risk of bias in the domain of self-fulfilling prophecy because death was either preceded by withdrawal of life-sustaining treatment or this information was not reported. The quality of evidence was not limited by inconsistency or indirectness but was limited by imprecision. Only one study found no significant impact of age on acute in-hospital mortality, and this study had methodologic limitations, primarily related to study participation and statistical reporting [34]. While the body of evidence was consistent in identifying older age as a predictor of acute in-hospital mortality in patients with cervical injury [35–38] and when patients with all levels of injury were included [39–41], the panel could not recommend the use of age alone as a reliable or moderately reliable predictor because, irrespective of age, the majority of individuals survive to hospital discharge. In one of the largest studies (n=3,389 patients with all levels and severity of injury), 528/668 patients (79%) ≥ 65 years survived to discharge [40].

Question: When counseling patients, family members, and/or surrogates of adults with acute tSCI, should comorbidities alone be considered a reliable predictor of acute in-hospital mortality?

- Recommendation: When counseling patients, family members, and/or surrogates of adults with acute tSCI, we suggest that comorbidities alone not be considered a reliable predictor of acute in-hospital mortality (weak recommendation, low quality evidence).
- Rationale: The body of evidence was downgraded for risk of bias, with various studies demonstrating potential bias in the QUIPS domains of study participation, prognostic factor measurement (assessment of comorbidities through retrospective chart review), confounding (potential impact of treatment factors), and self-fulfilling prophecy (withdrawal of life-sustaining treatment not considered or not reported). The quality of evidence was not limited by imprecision or indirectness, but inconsistency did downgrade the quality of evidence. The presence of two or more comorbid conditions compared to no comorbidities was associated with increased mortality in two large studies that included a total of more than 6,000 patients [39, 40]. However, even with ≥ 3 comorbidities, most patients in these two studies survived to hospital discharge (in-hospital mortality rate 16.9–22.8%). In one smaller study (n = 297) that utilized both the CCI and number of comorbidities, the number of comorbidities did not impact in-hospital mortality, although the study population included a large proportion (60.9%) of patients with AIS D SCI [42]. Across studies, the body of literature demonstrated substantial variation in study population and in prognostic factor measurement. The panel was unable to recommend the use of comorbidities as a reliable or moderately reliable predictor of mortality because most patients will survive despite the presence of multiple comorbidities.

Question: When counseling patients, family members, and/or surrogates of adults with acute tSCI, should concomitant injury alone be considered a reliable predictor of acute in-hospital mortality?

- Recommendation: When counseling patients, family members, and/or surrogates of adults with acute tSCI, we suggest that concomitant injury alone not be considered a reliable predictor of acute in-hospital mortality (weak recommendation; low quality evidence).
- Rationale: The body of evidence was downgraded for risk of bias, with various studies demonstrating potential bias in the QUIPS domains of prognostic factor measurement, confounding, statistical analysis and reporting, and self-fulfilling prophecy. Prognostic factor measurement varied between studies, including a count of body systems injured [40], total ISS [39, 41], and measures of the severity of associated TBI [35, 39, 41]. Most studies did not describe or control for the potential impact of treatment variables, descriptions of statistical analysis lacked detail, and the impact of withdrawal of life-sustaining treatment was not addressed. The quality of evidence was not limited by inconsistency or indirectness but was decreased by imprecision. Studies found increased risk of in-hospital mortality in patients with multisystem injury (hazard ratio 1.46–1.85) [40] and patients with higher total ISS [39, 41]. One study used the Trauma Score and ISS (TRISS) and found no significant association with acute in-hospital mortality [36]. Concomitant TBI was associated with higher acute in-hospital mortality, although TBI was defined differently across studies [35, 38, 39, 41]. However, in those studies in which mortality rates were separately reported, more than 80% of patients with TBI or other multisystem trauma survived to hospital discharge [40, 41]. The panel was unable to recommend the use of concomitant injury alone as a reliable or moderately reliable predictor of mortality because most patients will survive despite the presence of concomitant injuries.

Question: When counseling patients, family members, and/or surrogates of adults with acute tSCI, should NLI alone be considered a reliable predictor of acute in-hospital mortality?

- Recommendation: When counseling patients, family members, and/or surrogates of patients with acute tSCI, we suggest that NLI alone not be considered a reliable predictor of acute in-hospital mortality. (weak recommendation; very low quality evidence)
- Rationale: The body of evidence was downgraded for risk of bias, with various studies demonstrating potential bias in the QUIPS domains of study participation, prognostic factor measurement, study confounding, statistical analysis and reporting, and self-fulfilling prophecy. In addition, several studies included limited or no information about the timing of prognostic factor measurement and decisions about withdrawal of life-sustaining treatment. The quality of evidence was not limited by indirectness but was limited by inconsistency and imprecision. Most studies included only patients with cervical injuries. In the only study that included patients with all levels of injury, the majority of the population (83.5% of 6,827 patients) had cervical injury [41]. There were no significant differences in mortality based on NLI when comparing cervical, thoracic, or cauda equina to lumbar levels of injury [41]. Among studies focused on cervical injury, mortality was higher in patients with upper cervical injury, which was defined as C4 or higher in two studies [34, 35] and C5 or higher in the largest study [43]. In contrast, Martin and colleagues found no significant difference in mortality between patients with C1–4 and C5–8 injuries [36]. Across all studies, the vast majority of patients (approximately 80–90%) survived to hospital discharge. Additional limitations that favor not considering the predictor include the potential for withdrawal bias among patients with cervical injury and the likelihood that patients with complete injury at C1 or C2 do not survive to reach the hospital.

Question: When counseling patients, family members, and/or surrogates of adults with acute tSCI, should severity of injury (complete versus incomplete) alone be considered a reliable predictor of acute in-hospital mortality?

- Recommendation: When counseling patients, family members, and/or surrogates of patients with acute tSCI, we suggest that severity of injury (complete versus incomplete) alone not be considered a reliable predictor of acute in-hospital mortality (weak recommendation; low quality evidence).
- Rationale: The body of evidence was downgraded for risk of bias, with various studies demonstrating potential bias in the QUIPS domains of study participation, study attrition, prognostic factor measurement, study confounding, statistical analysis and reporting, and self-fulfilling prophecy. In several studies, the population was either selective or was poorly described, and some studies did not consider treatment effect, the impact of polytrauma, or withdrawal of life-sustaining treatments. Limited descriptions of statistical analysis also contributed to risk of bias. The quality of evidence was not limited by inconsistency or indirectness, but was impacted by imprecision with wide confidence intervals in one large study [43]. Mortality risk appears increased with complete injury when compared to incomplete injury, especially at the cervical level, but variability in comparison groups and statistical analysis make the risk difficult to quantify [34, 36, 38–41, 43]. Even among patients with complete cervical injury, early survival is consistently reported to exceed 75% [34, 38, 39, 43].

There was insufficient evidence to provide a recommendation on the use of MRI findings as a predictor of in-hospital mortality.

### Outcome: Long-Term Mortality (Cumulative, Measured After Discharge)

Question: When counseling patients, family members, and/or surrogates of adults with acute tSCI, should older age at the time of injury alone be considered a reliable predictor of long-term mortality (cumulative mortality measured at any time point after hospital discharge)?

- Recommendation: When counseling patients, family members, and/or surrogates of patients with acute tSCI, we suggest increased age at time of injury alone not be considered a reliable predictor of long-term mortality (cumulative mortality measured at any time point after hospital discharge). (weak recommendation; low quality evidence)
- Rationale: The body of evidence was downgraded for risk of bias, with various studies demonstrating potential bias in the QUIPS domains of study participation, outcome measurement, confounding, statistical analysis and reporting, and self-fulfilling prophecy. The quality of evidence was not limited by inconsistency or indirectness, but was limited by imprecision with wide confidence intervals throughout. While there is low quality evidence that suggests that the risk of longer-term all-cause mortality (at least 1 year after tSCI) increases with age [44–48], the age at which this increase is seen varies substantially between studies, follow-up periods vary, confidence intervals are wide, and comparator groups vary. Survival at a year or more postinjury remains > 50% for almost all age groups [44, 45, 47], although one study [45] reported a five-year survival of 40.4% among those individuals aged 75 or older. With one exception [46], acute in-hospital mortality was included in the cumulative mortality rates reported at later intervals.

Question: When counseling patients, family members, and/or surrogates of adults with acute tSCI, should comorbidities alone be considered a reliable predictor of long-term mortality (cumulative mortality measured at any time point after hospital discharge)?

- Recommendation: When counseling patients, family members, and/or surrogates of patients with acute tSCI, we suggest that comorbidities alone not be considered a reliable predictor of long-term mortality (cumulative mortality measured at any time point after hospital discharge; weak recommendation; low quality evidence).
- Rationale: Three studies [45, 46, 49] evaluated the predictive value of comorbid conditions at the time of acute hospitalization for long-term cumulative mortality. Moderate risk of bias was present, with potential bias in at least one study for all QUIPS domains. The quality of evidence was not limited by imprecision or indirectness but was limited by inconsistency. One study assessed all-cause mortality 5 years post injury in patients with all levels and varying severity of tSCI, and found no correlation with CCI during acute hospitalization [45]. Similarly, Esmoris-Arijón and colleagues (2021) found no association between CCI during acute hospitalization and death at 1 year [49]. The third and largest (n=2,685) study evaluated the association between number of comorbidities and mortality after discharge from acute hospitalization. This study followed patients for variable periods (longest follow-up > 10 years) and found that patients with two or more comorbid conditions were at increased risk for death. However, 84% of patients remained alive at last follow-up [46].

Question: When counseling patients, family members, and/or surrogates of adults with acute tSCI, should concomitant injury alone be considered a reliable predictor of long-term mortality (cumulative mortality measured at any time point after hospital discharge)?

- Recommendation: When counseling patients, family members, and/or surrogates of patients with acute tSCI, we suggest that concomitant injury alone not be considered a reliable predictor of long-term mortality (cumulative mortality measured at any time point after hospital discharge; weak recommendation; moderate quality evidence).
- Rationale: The evidence was downgraded for risk of bias in the QUIPS domains of study participation, prognostic factor measurement, confounding, and self-fulfilling prophecy. The quality of evidence was not limited by inconsistency, imprecision, or indirectness. All studies reported cumulative mortality, encompassing in-hospital and post-discharge mortality. Concomitant injury was defined by chest trauma [44], ISS [45], or GCS [45, 47], and patient population also varied by level and severity of injury. None found a significant impact of concomitant injury on long-term survival after tSCI.

Question: When counseling patients, family members, and/or surrogates of adults with acute tSCI, should NLI alone be considered a reliable predictor of long-term mortality (cumulative mortality measured at any time point after hospital discharge)?

- Recommendation: When counseling patients, family members, and/or surrogates of patients with acute tSCI, we suggest that NLI alone not be considered a reliable predictor of long-term mortality (cumulative mortality measured at any time point after hospital discharge). (weak recommendation; very low quality evidence)
- Rationale: Four studies addressed the impact of NLI on long-term outcomes, with follow-up intervals varying from 1 year to almost 12 years. The body of evidence was downgraded for risk of bias, with various studies demonstrating potential bias in the QUIPS domains of study participation, prognostic factor measurement, study confounding, statistical analysis and reporting, and self-fulfilling prophecy. The quality of evidence was not limited by indirectness but was limited by inconsistency and imprecision. One study reported 5-year mortality for 426 patients with cervical, thoracic, and lumbar injury of variable severity and found no significant impact of NLI on multivariate analysis [45]. Another found no significant difference in cumulative mortality at 1 year for cervical versus thoracolumbar injury [47]. The remaining two studies found increased mortality in patients with upper cervical injury (C1-C4); the comparison group was patients with lower cervical injury in one study [44] and all levels of injury in the other [46]. Even among patients with injuries at C4 or above, approximately 60–70% were still alive 8 to 10 years post injury [44, 46], limiting the utility of NLI as a predictor of long-term mortality.

Question: When counseling patients, family members, and/or surrogates of adults with acute tSCI, should severity of injury alone be considered a reliable predictor of long-term mortality (cumulative mortality measured at any time point after hospital discharge)?

- Recommendation: When counseling patients, family members, and/or surrogates of patients with acute tSCI, we suggest that severity of injury alone (defined by complete versus incomplete injury or by ASIA motor score) not be considered a reliable predictor of long-term mortality (cumulative mortality measured at any time point after hospital discharge; weak recommendation; low quality evidence).
- Rationale: The body of evidence was downgraded for risk of bias, with various studies demonstrating potential bias in the QUIPS domains of study participation, prognostic factor assessment, study confounding, statistical analysis and reporting, and self-fulfilling prophecy. The quality of evidence was not limited by inconsistency or indirectness but was impacted by imprecision. Although increased severity of injury (defined by lower ASIA motor score or worse AIS/Frankel grade) appeared to be associated with increased mortality risk, the evidence was limited by variation in study methodology, and lack of information regarding the timing of assessment of injury severity. The largest study reported a hazard ratio of 0.3–0.6 for incomplete injury (Frankel grades B-E) versus complete injury (Frankel grade A) with overall mortality (across all grades) of 16.2% [46]. Although follow-up intervals varied and severity of injury did impact mortality, survival rates at final follow-up remained above 60 percent across studies even for patients with the most severe injuries. Casper et al. (2018) grouped patients by ASIA motor score and found that lower motor scores were associated with higher mortality; however, 5-year survival was 68.2% even among the group with the most severe injury (ASIA motor score 0–20) [45].

There was insufficient evidence to provide a recommendation on the use of MRI findings as a predictor of long-term mortality.

### Outcome: Functional Outcome (at Discharge from Rehabilitation or Beyond)

Question: When counseling patients, family members, and/or surrogates of adults with acute tSCI, should older age at time of injury alone be considered a reliable predictor of worse functional outcome at discharge from rehabilitation or beyond?

- Recommendation: When counseling patients, family members, and/or surrogates of patients with acute tSCI, we suggest that age alone not be considered a reliable predictor of functional outcome at 1-year follow-up (weak recommendation; moderate quality evidence).
- Rationale: Functional outcome was assessed with the FIM score in studies that met our criteria. Evidence was downgraded for the overall risk of bias with potential bias in the QUIPS domains of participation, study attrition, confounding, statistical analysis and reporting and self-fulfilling prophecy. The quality of evidence was not limited by inconsistency, indirectness, or imprecision. Available data from four large prospective databases suggested that age at time of the injury was independently and inversely associated with functional outcome at 1 year. Age could not be considered a reliable, or moderately reliable predictor, because these effects were weak [28, 47, 50, 51]—albeit statistically significant—and an age threshold that reliably predicted poor outcome could not be identified. In one study, advanced age impacted functional outcome and recovery after tSCI as assessed by the motor score of the FIM. This effect varied across the spectrum of injury severity. Potential for improvement generally diminished with completeness or incompleteness of the injury. Age was particularly relevant for AIS grade B/C patients, with patients < 65 years demonstrating better functional outcomes [51].

Question: When counseling patients, family members, and/or surrogates of adults with acute tSCI, should the NLI alone be considered a reliable predictor of functional outcome at discharge from rehabilitation or beyond?

- Recommendation: When counseling patients, family members, and/or surrogates of patients with acute tSCI, we suggest that NLI alone not be considered a reliable predictor of poor functional outcome at 1-year follow-up. However, patients and surrogates should be counseled that a higher level of injury is associated with more functional deficits and greater dependence (weak recommendation; low quality evidence).
- Rationale: The body of evidence was downgraded for risk of bias, with various studies demonstrating potential bias in the QUIPS domains of study attrition, confounding, statistical analysis and reporting, and self-fulfilling prophecy. Imprecision was present. In a multivariate analysis thoracolumbar injuries independently predicted better functional outcome than cervical injuries [51]. The study was limited however by a relatively low proportion (<10%) of thoracic and lumbar SCI. In thoracic SCI the NLI based on the sensory exam was also an independent predictor of functional outcome. The analysis of 400 patients enrolled in the SCIMS database revealed better FIM motor scores at 1-year follow-up in patients with low (T10–T12) as opposed to high (T2–T9) NLI [28]. This predictor could not be recommended as a reliable (or moderately reliable) predictor *in isolation* because a substantial number of patients with higher NLI with mitigating factors such as incomplete injury achieve functional independence.

Question: When counseling patients, family members, and/or surrogates of adults with acute tSCI, should the initial severity of injury as measured by the ASIA impairment score alone be considered a reliable predictor of functional outcome at discharge from rehabilitation or beyond?

- Recommendation: When counseling patients, family members, and/or surrogates of patients with acute tSCI, we suggest that initial severity of injury as measured by the ASIA impairment score alone not be considered a reliable predictor for future motor outcome at 1-year follow-up. However, patients and surrogates should be counseled that motor incomplete injury is associated with a higher probability of functional improvement (weak recommendation; moderate quality evidence).
- Rationale: The body of evidence was downgraded for risk of bias, with various studies demonstrating potential bias in the QUIPS domains of study attrition, confounding, statistical analysis and reporting, and self-fulfilling prophecy. The quality of evidence was not limited by inconsistency, indirectness, or imprecision. Evidence was from prospective registries and study data sets revealed a strong relationship between initial ASIA grade and functional outcome as measured by the motor score of the FIM. As independent predictors of functional outcome, the AIS and NLI are most consistently included in clinical prediction models. Patients with motor complete injuries (AIS A and B) were least likely to achieve functional independence, compared with patients with AIS C and D injuries [50, 51]. As previously outlined, these studies primarily included patients with cervical tSCI, about half with motor complete injuries. However, an analysis by Lee et al. (2016) confirmed these results in a population with thoracic tSCI, in which 90% of patients had motor complete injuries [28]. This predictor could not be recommended as a reliable (or moderately reliable) predictor *in isolation* because a substantial number of patients with motor complete injuries demonstrate AIS grade conversion and variable functional independence at long-term follow-up.

There was insufficient evidence to provide a recommendation on comorbidities as a predictor of functional outcome. Data from individual studies of concomitant injuries and MRI as predictors of functional outcome were also insufficient to support recommendations. A summary of these studies is available in Supplementary Appendix 4.

### Outcome: AIS Improvement (Conversion) at Discharge from Rehabilitation or Beyond

Question: When counseling patients, family members, and/or surrogates of adults with acute tSCI, should age at the time of injury alone be considered a reliable predictor of AIS improvement (conversion) assessed at discharge from rehabilitation or beyond?

- Recommendation: When counseling patients, family members, and/or surrogates of patients with acute tSCI, we suggest age at the time of injury alone not be considered a reliable predictor of AIS conversion assessed at 12–24 months follow-up (weak recommendation; moderate quality evidence).
- Rationale: The body of evidence was downgraded for risk of bias, with various studies demonstrating at least moderate bias in the QUIPS domains of study participation, study attrition, prognostic factor assessment, outcome measurement, study confounding, statistical analysis and reporting, and self-fulfilling prophecy. Three studies met inclusion criteria. In two studies, no effect of age at the time of injury on the rate of AIS conversion was observed. In a study of 14,433 patients with tSCI from the SCIMS database, neither age alone, nor the interaction of gender and age, was a significant predictor of AIS conversion [52, 53]. In one study of 57 patients with cervical tSCI who underwent surgical management, the median age was 37 years in the group that demonstrated AIS conversion, compared to a median age of 64 years in the group that did not [54].

Question: When counseling patients, family members, and/or surrogates of adults with acute tSCI, should pathological findings on MRI be considered a reliable predictor of AIS conversion at discharge from rehabilitation or beyond?

- Recommendation: When counseling patients, family members, and/or surrogates of patients with acute tSCI, we suggest that the absence of pathological findings on MRI be considered a moderately reliable predictor of AIS conversion at 6–12 months follow-up (weak recommendation; low quality evidence).
- Rationale: The body of evidence was downgraded for risk of bias, with various studies demonstrating at least moderate bias in the QUIPS domains of study participation and attrition, prognostic factor measurement, confounding, and self-fulfilling prophecy. Imprecision was present. In one study of 86 patients with cervical tSCI (AIS grades B-D), with improvement of at least one AIS grade in 77% of all patients at 12-month follow-up, intramedullary edema >36 mm and facet dislocation on MRI were independent predictors of the absence of AIS conversion regardless of initial AIS grade [55]. In another study, stepwise multiple logistic regression analysis demonstrated a 5% reduction in the probability of AIS conversion with each mm increment in intramedullary lesion length [56]. In a group of 55 patients who received definitive treatment for subaxial cervical fracture dislocations, AIS conversion was observed in 54% at follow-up. Maximal spinal cord compression >55.8% on MRI was an independent predictor of the absence of AIS conversion in this study [57].

Question: When counseling patients, family members, and/or surrogates of adults with acute tSCI, should the NLI be considered a reliable predictor of AIS conversion at discharge from rehabilitation or beyond?

- Recommendation: When counseling patients, family members, and/or surrogates of patients with acute tSCI, we suggest that NLI be considered a moderately reliable predictor of AIS conversion at 6–12 months follow-up. Patients and surrogates should be counseled that an improvement in AIS is more likely following lumbar and thoracolumbar injury than cervical or thoracic injury (weak recommendation; moderate quality evidence).
- Rationale: The body of evidence was downgraded for risk of bias, with various studies demonstrating at least moderate bias in the QUIPS domains of study participation and attrition, prognostic factor and outcome measurement, confounding, statistical analysis and reporting, and self-fulfilling prophecy. In a retrospective study of 931 patients with motor complete (AIS A/B) tSCI NLI was an independent predictor of AIS improvement at 6–12 months. Patients with injury at the conus or below (lumbar) were more likely to demonstrate an improvement in AIS than patients with cervical or thoracic tSCI. [58]. In a study of 95 patients with thoracic and thoracolumbar injuries, an AIS conversion rate of 92.9% was observed following lumbar tSCI (conus, L1-S5) versus 22.4% for thoracic and thoracolumbar tSCI [59]. In a multivariate analysis of 86 surgically treated patients, compared with patients with cervical tSCI, patients with thoracolumbar and lumbar injury were significantly more likely to demonstrate AIS improvement (odds ratio [OR] 3.9), whereas patients with thoracic injury were less likely to improve (OR 0.5) [60].

Question: When counseling patients, family members, and/or surrogates of adults with acute tSCI, should the initial severity of injury as measured by the ASIA impairment score be considered a reliable predictor of AIS conversion at discharge from rehabilitation or beyond?

- Recommendation: When counseling patients, family members, and/or surrogates of patients with acute tSCI, we suggest that severity of injury as assessed by the initial ASIA impairment score be considered a moderately reliable predictor of AIS conversion at 6–12 months follow-up. Patients and surrogates should be counseled that an improvement in AIS is more likely in the presence of incomplete injury (weak recommendation; low quality evidence).
- Rationale: The body of evidence was downgraded for risk of bias, with various studies demonstrating at least moderate potential bias in the QUIPS domains of study participation and attrition, prognostic factor and outcome measurement, confounding, statistical analysis and reporting, and self-fulfilling prophecy. Imprecision was present. The body of evidence was consistent in demonstrating an independent association between severity of SCI as assessed by the initial ASIA impairment score and AIS conversion. Less than one third of patients with AIS A injuries demonstrate improvement at 1 year. AIS conversion is 4 to 8-fold more likely with AIS B–D injuries compared with AIS A injuries [53, 54, 58]. In a study of 931 patients with motor complete tSCI (AIS A/B) 21% of all patients with initial AIS A showed neurological improvement at 1-year follow-up compared to 69% with initial AIS B [58]. Among 86 patients with incomplete tSCI (AIS B–D), AIS improved by one or more grades in nearly 75%. Although incomplete injury was an independent predictor of AIS improvement in this study, AIS D was present in more than half of these patients at the time of initial evaluation [55]. In a study of 58 patients operated for thoracic or thoracolumbar tSCI, AIS conversion at 1-year follow-up occurred in 35% of patients with initial AIS A, 82% of patients with AIS B, 80% of patients with AIS C and 90% of patients with AIS D [61]. In a study of 86 patients with tSCI who underwent surgery only AIS B (OR 4.3, 95%CI 1.2–15.4) and AIS D (OR 5.2, 1.2–21.6) were independent predictors of AIS improvement [60].

There was insufficient evidence to provide a recommendation on concomitant injury as a predictor of AIS conversion. Data from individual studies of the predictive value of comorbidities for AIS conversion were also insufficient to support recommendations. A summary of these studies is available in Supplementary Appendix 4.

### Outcome: Independent Ambulation (at Discharge from Rehabilitation or Beyond)

The body of evidence for individual predictors of independent ambulation was insufficient to support recommendations. A summary of individual studies is available in Supplementary Appendix 4*.*

## Recommendations: Clinical Prediction Models

One model, the DCPR for ambulation, met criteria for inclusion. The DCPR predicts the probability of independent ambulation at 1 year post injury using information available during early management of tSCI. Tables 2 and 3 provide more information about DCPR score calculation and use.

### Outcome: Independent Ambulation (at Discharge from Rehabilitation or Beyond)

Question: When counseling patients, family members, and/or surrogates of adults with acute tSCI, should the *DCPR for ambulation,* with neurological assessment performed within 15 days following injury, be considered a reliable predictor of the ability to walk independently at discharge from rehabilitation or beyond?

- Recommendation: When counseling patients, family members, and/or surrogates of patients with acute tSCI, we suggest the Dutch Clinical Prediction Rule for ambulation (DCPR)*,* with neurological assessment performed within 15 days following injury, be considered a moderately reliable predictor of the ability to walk 1-year following injury (weak recommendation; moderate quality evidence).
- Rationale: The body of evidence was downgraded for risk of bias, with various studies demonstrating bias in the Prediction model Risk Of Bias Assessmnent Tool (PROBAST) domains of analysis and self-fulfilling prophecy.
- The DCPR was introduced in 2011 and is based on a patient sample set from the EMSCI. It was first validated internally and then externally in various international cohorts [19–22]. Excellent discrimination (AUC >0.95) was reported in most studies [18, 20]. Simplifying the rule by leaving out age (AUC 0.94) or leaving out one motor score (S1) and one sensory score (L3) (AUC 0.87) may yield acceptable discrimination [21]. Some recent studies suggest that prediction accuracy may vary based on injury severity (ASIA A+D > B+C) [22, 62]. Clinical utility may be greatest in patients with AIS B and C injuries since improvement to functional independence is inherently unlikely with AIS A injuries and inherently likely with AIS D injuries. Other studies suggest superior model performance with a lower age threshold than 65 years [63]. In a validation study using the SCIMS database dichotomization of age at 50 years yielded superior model performance, with both the original DCPR and a simplified (“3-variable”) version [22].

## Future Directions

While individual clinical variables may independently predict an outcome in multivariate analysis, they rarely achieve the predictive accuracy necessary to serve as the sole basis for clinical neuroprognostication. A multimodal approach is therefore essential. The interaction of age and comorbidities, probably best described as “frailty,” will need better scientific exploration and consensus on descriptors (“frailty indices”) used. In the setting of tSCI it is also essential to consider multidimensional (motor, sensory, cognitive, socioeconomic, etc.), patient-centered functional outcomes, as well as the potential interaction between social determinants of health and outcomes. Clinical prediction models incorporate multiple independent predictors to improve prognostic accuracy, but only a limited number of models exist. While models such as the DCPR may benefit from further modifications and validation to improve predictive accuracy, they provide a reasonable estimate of the likelihood of meaningful outcomes, such as a return to ambulation. These estimates allow patients and families to set realistic expectations and plan for the future.

The core of tSCI research is a standardized approach to clinical examination and documentation through consistent utilization of the ISNCSCI scoring system. This system is constantly reviewed and refined. New items, such as ZPP, may substantially add to future research endeavors. The impact of consistent use of the ISNCSCI scoring system on the scientific value of the published evidence cannot be overemphasized because it provides a standardized nomenclature and thus the ability to compare similar populations across studies. The impact of timing of assessment on the accuracy of outcome prediction requires additional research.

Most of the evidence used as the basis for recommendations within these guidelines was derived from multicenter registries and data sets. Statistical value increases with larger samples, and accurate, detailed descriptions of the population. The use of predefined common data elements (CDEs) in published studies is crucial [64].

Modern imaging technology (MRI, tractography, spectroscopy) is increasingly a standard in tSCI care, and will ultimately enhance multimodal prognostication. Advanced neuromonitoring of parameters such as intraspinal pressure, perfusion and oxygenation is currently not in routine clinical use but may contribute to both management and prognostication in the future. Similarly, novel biomarkers from serum and cerebrospinal fluid may assist with prognostication and provide insights into novel therapeutic options in the future. Further preclinical and clinical biomarker research is therefore necessary.

While these guidelines focus on a limited range of outcomes, such as long-term independent ambulation, patients and families value a wide range of bodily functions and self-care activities. Nonspecific outcomes such as AIS grade conversion do not translate linearly to improved function and have limited meaning to patients and families. In a survey of more than 600 patients, recovery of arm and hand function was most important to quadriplegics, while recovery of sexual function was of highest priority to paraplegics. Both ranked improvement of bowel and bladder function equally high [65]. The body of evidence for predictors of recovery of these functions is especially scarce. Future research should focus on these additional patient-centered outcomes.

Therapeutic interventions were outside the scope of these guidelines, and our recommendations assume provision of the standard of care (best medical practice). However, the treatment of tSCI is heterogeneous, and best medical practice is constantly evolving. As an example, early surgical decompression of the spinal cord has received greater emphasis more recently, in addition to restoration of spinal stability. Future prognostication studies should consistently include critical aspects of tSCI treatment, such as early surgical decompression, as variables in multivariate analysis and, where appropriate, in prediction models.

Based on the most common study limitations identified in our systematic review, future studies should consider the following general principles:

1. Clear description of the study population and a predetermined time point of outcome assessment is necessary to limit study bias related to patient participation and attrition.
2. Specification of the timing of neurological assessment is essential for studies evaluating the predictive accuracy of NLI and AIS grade.
3. As a consequence of the heterogeneity of tSCI, only the largest registries provide a sufficient sample size to analyze predictors across all levels and severity of injury. Participation in multicenter tSCI registries should be encouraged.
4. The use of logical patient groupings (for example, separating AIS A, AIS B/C and AIS D, and cervical/high thoracic NLI from low thoracic/lumbar NLI for analysis), will allow researchers to generate clinically meaningful information.
5. Withdrawal of life-sustaining treatment is thought to be uncommon following tSCI, but the true frequency is unknown because most studies do not report this information. Including information about withdrawal of life-sustaining treatment will decrease potential bias associated with self-fulfilling prophecy.
6. Consistent use of validated tools for measurement of both prognostic factor and outcomes will substantially improve the quality of evidence for neuroprognostication after tSCI. Use of the measurement tools outlined in the CDEs for spinal cord injury is strongly encouraged [64].
7. In addition to standardized study design as outlined above, power calculations and appropriate statistical analysis should be performed.

## Conclusions

**Fig. 2:**
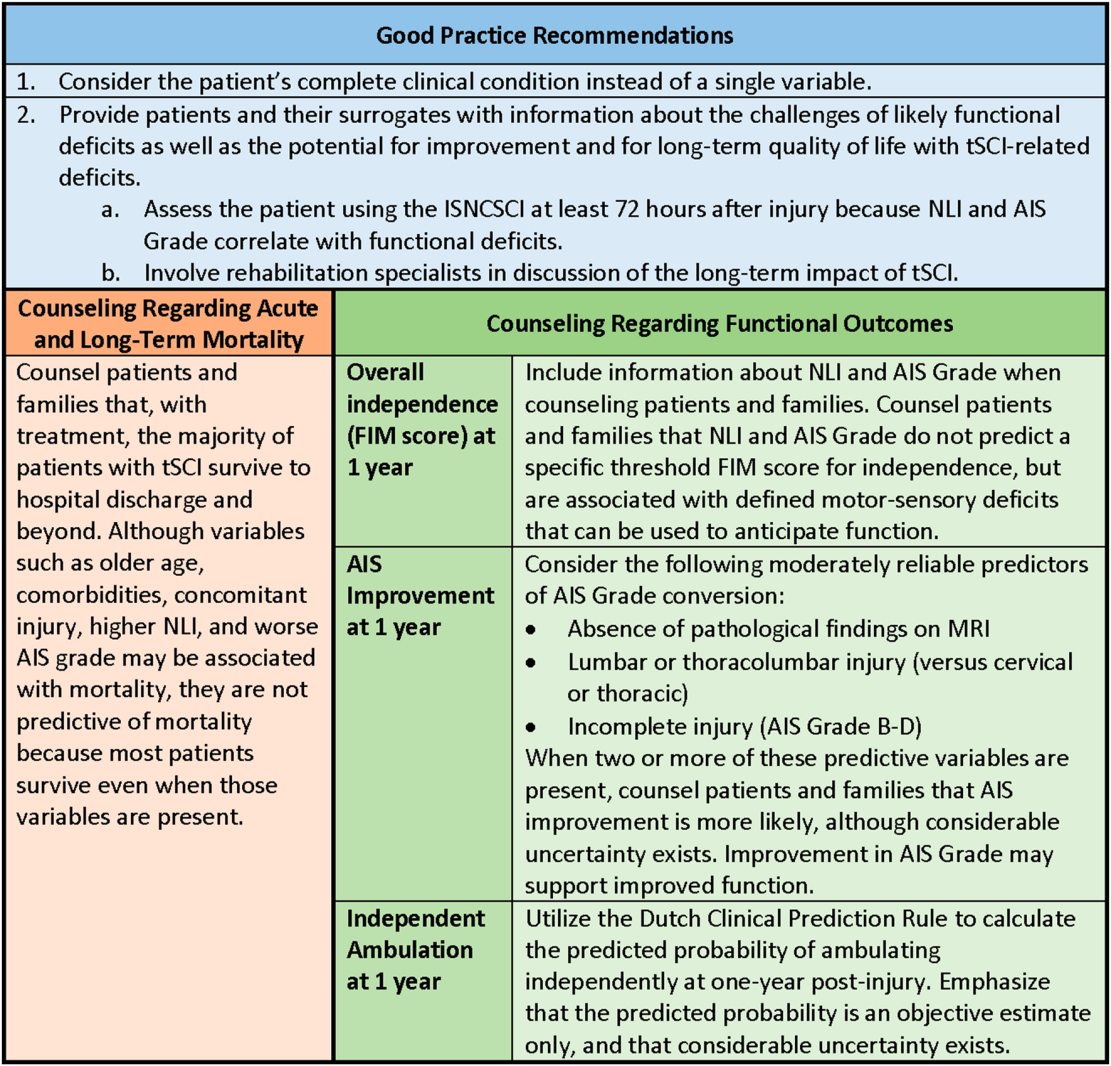
Suggested approach to neuroprognostication after traumatic spinal cord injury (tSCI). AIS, American Spinal Injury Association Impairment Scale, FIM, Functional Independence Measure, ISNCSCI, International Standards for the Neurological Classification of Spinal Cord Injury, NLI, Neurological Level of Injury

These guidelines provide recommendations on the use of predictors of mortality as well as functional outcome in the context of counseling adult patients with tSCI and their surrogates. Recommendations are summarized in Table 5. A suggested approach to neuroprognostication after tSCI is summarized in Fig. 2. Three predictors (absence of pathologic findings on MRI, NLI, and injury severity) were considered moderately reliable for the prediction of AIS conversion at 1-year follow-up. A clinical prediction model, the DCPR, was considered moderately reliable for the prediction of independent ambulation at 1-year follow-up. Future development of additional biomarkers and models will help enhance the field of neuroprognostication in tSCI.

**Table 5:** Summary of recommendations

		Outcomes (all outcomes except mortality measured at discharge from rehabilitation or beyond)					
		Mortality, acute in-hospital	Mortality, measured after discharge, cumulative	Functional outcome (SCIM/FIM)	AIS conversion	Ambulation	Bladder and bowel function
Clinical variables	Age	not reliable	not reliable	not reliable	not reliable	insufficient data	no data
	Comorbidities	not reliable	not reliable	no data	insufficient data	no data	no data
	Concomitant injury	not reliable	not reliable	insufficient data	no data	no data	no data
	MRI	no data	no data	insufficient data	moderately reliable	no data	no data
	NLI	not reliable	not reliable	not reliable	moderately reliable	insufficient data	no data
	Severity of SCI	not reliable	not reliable	not reliable	moderately reliable	insufficient data	no data
Predictive model	DCPR	not applicable	not applicable	not applicable	not applicable	moderately reliable	not applicable

No data: No studies were identified that met criteria for inclusion

Insufficient data: One study met criteria for inclusion; body of literature inadequate to make a recommendation based on the a priori methodological agreements of the panel

DCPR, Dutch Clinical Prediction Rule, FIM, Functional Independence Measure, MRI, magnetic resonance imaging, NLI, neurological level of injury, SCIM, Spinal Cord Independence Measure, SCI, spinal cord injury

### Supplementary Information

Below is the link to the electronic supplementary material.

Supplementary file 1 (DOCX 38 KB)

Supplementary file 2 (DOCX 14 KB)

Supplementary file 3 (XLSX 65 KB)

Supplementary file 4 (DOCX 42 KB)

Supplementary file 5 (DOCX 14 KB)

Supplementary file 6 (DOCX 14 KB)

Supplementary file 7 (DOCX 14 KB)

Supplementary file 8 (DOCX 23 KB)
