# Supplementary material for: Guidelines for neuroprognostication in adults with traumatic spinal cord injury
Source: Neurocrit Care. 2023 Nov 13;40(2):415–37. doi: 10.1007/s12028-023-01845-8 (PMC10959804; doi:10.1007/s12028-023-01845-8)
Supplement: Supplementary file 1 — (DOCX 38 KB) [file 12028_2023_1845_MOESM1_ESM.docx]

**Supplementary appendix 1**

**Neurocritical Care Society (NCS) and Deutsche Gesellschaft für Neurointensivmedizin (DGNI) Neuroprognostication guidelines: Methodology**

**How to use these guidelines**

These guidelines provide recommendations on the reliability of selected individual clinical variables and prediction models (both hereafter referred to as “predictors”). These predictors have been categorized as reliable, moderately reliable, or not reliable. We based this categorization on the GRADE criteria supporting the use of the predictor in the context of counseling, as well as effect size. This categorization led to the recommendation to either use or not use the predictor to formulate a prognosis, caveats to the use of the predictor, and suggested language during counseling (**Predictor categorization tables 1 & 2,** in this supplementary document, below).

A key distinction exists between a *reliable* predictor of outcome in the context of counseling surrogates of patients requiring neurocritical care and an *independent* predictor of outcome. An independent predictor fulfills one criterion—a statistically significant association with the outcome of interest in an appropriately conducted multivariate analysis. In clinical practice, independent predictors of outcome may be used in risk stratification, selection of patients for targeted treatment (such as chemotherapy regimens for cancer), or as building blocks of clinical prediction models.[1, 2] A reliable predictor in the context of counseling patients requiring neurocritical care or their family members must be independent, but also fulfil other criteria as described in the “Effect size (Predictor accuracy)” and “Evidence to Recommendation (EtoR)” sections.

**Reliable** predictors, for the purposes of these guidelines, may be used to formulate a prognosis when the appropriate clinical context is present in the absence of potential confounders. These are predictors with clear, actionable thresholds or clinical/radiographic definitions and a low rate of error in prediction of poor outcomes, with at least moderate certainty in the body of evidence using GRADE criteria. When the prognosis is formulated on the basis of one or more reliable predictors, the clinician may describe the outcome as “very likely” during counseling. Given the inherent limitations in neuroprognostication research, the clinician must nevertheless acknowledge the presence of uncertainty—albeit low—in the prognosis.

**Moderately reliable** predictors may be used for prognostication *only* when additional reliable or moderately reliable predictors are present, in addition to the appropriate clinical context as specified above. These are also predictors with clear, actionable thresholds or clinical/radiographic definitions and a low rate of error in prediction of poor outcomes, but with lower certainty in the body of evidence using GRADE criteria, typically as a result of smaller (imprecision) and/ or fewer studies. When the prognosis is formulated on the basis of multiple moderately reliable predictors, the clinician may describe the outcome as “likely” during counseling, but must acknowledge “substantial” uncertainty in the prognosis.

While the panelists recognize that those predictors that do not meet the criteria to be described as reliable or moderately reliable are often used by clinicians in formulating their subjective impressions of prognosis, they have nevertheless been deemed **not reliable** for the purposes of these guidelines and cannot be formally recommended for prognostication on their own. Variables deemed not reliable however may be a component of reliable or moderately reliable prediction models.

**Guideline panel**

The NCS and DGNI recruited 20 professionals in neurocritical care, nursing, and pharmacy to create a panel of experts for the neurocritical condition considered; including a NCS-assigned GRADE methodologist in 2018. The guideline panel consisted of two to four content experts who served as the primary authors for each disease, an affected patient or family member who served as the public representative, and the content experts of the other seven disease states which included the two chairs of the entire neuroprognostication guidelines. The inclusion of neurointensivists, neurosurgeons, advanced practice providers, pharmacists, and a neuropalliative care expert on the guideline panel created a multidisciplinary collaboration. A statistician-epidemiologist with expertise in neuroprognostication and medical decision making served as a consultant. The panel convened in monthly video-conferences, with in-person meetings at two consecutive annual meetings of the NCS and one annual meeting of DGNI. Additional small group video and telephone meetings occurred ad hoc among content experts, the chairs, the GRADE expert, and the public representative. Recommendations were voted on using online survey software (Google Forms, Google Inc., Mountain View, California, United States). Panelists were required to disclose all potential conflicts of interest prior to participation. Potential conflicts were reviewed by the co-chairs of the panel, as well as the NCS guidelines committee, and managed in accordance with policies of NCS.

**Selection of guideline questions**

An initial Patient, Intervention, Comparison, Outcome and Time (PICOTS) question was framed to help identify candidate predictors: *“What are the reliable predictors (prognostic factors, variables, tests, scores, and multivariable models), prior to and on admission as well as during the hospital course to predict patient outcome at different follow up time points following each specific disease?”*. A professional librarian executed a comprehensive literature search using search criteria appropriate to this initial question. Since this search was expected to generate a large volume of articles, additional criteria were used to select studies with a focus on reliability of predictors and appropriate outcomes. These criteria are described below in the “systematic review methodology” section. The content experts reviewed the full-text articles that fulfilled selection criteria. Candidate predictors were then selected by these experts and confirmed by the panel, based on clinical relevance, availability in daily clinical practice, AND the presence of an appropriate body of literature. Candidate predictors and prediction models were considered “clinically relevant” if, in the subjective opinion of the content experts and guideline chairs, the predictor or components of the prediction models were accessible to clinicians; although universal availability was not required AND likely to be considered by clinicians while formulating a prognosis. An appropriate body of literature was considered present for any predictor that fulfilled two criteria—1) evaluated in at least two published studies that included a minimum of 35-100 subjects (number of patients required was dependent on the disease state: 35 for Gullain-Barré Syndrome; 50 for spinal cord injury and status epilepticus; 100 for cardiac arrest, traumatic brain injury, acute ischemic stroke, intracerebral hemorrhage, and subarachnoid hemorrhage) and 2) established as an independent predictor in a multivariate analysis. An appropriate body of literature was considered present for clinical prediction models with at least one external validation study of at least 35-100 patients (dependent on the disease state as above) in addition to the initial report on development of the model (also with a minimum of 35-100 patients).

**Selection of outcomes**

Outcomes relevant to the question and each disease were identified by the panel, then rated by the primary content experts and the public representative. The outcomes were rated using the GRADE 1-9 scale and the score averaged. An outcome with an average score of greater than 6 was considered “critical” and included in the evaluation. The range of outcomes and the time period of outcome assessment was specific for each disease. The only outcomes included at the time of discharge from the acute care facility were mortality at discharge and beyond for most disease states and the need for mechanical ventilation with 14 days of onset of Guillain-Barré-Syndrome. Other outcomes considered were required to be assessed after a disease-specific longer time period for recovery and beyond. While a longer duration from time of injury to outcome assessment is ideal to capture the entirety of functional recovery, this prioritization may result in loss to follow-up. Significant loss to follow-up in observational studies may result in a selection bias, based on the patients most likely to respond or return to the index hospital for further medical care.

**Systematic review methodology**

Since the literature on prognosis was expected to be heterogenous, a narrative systematic review was performed. As described earlier, the initial librarian search string was appropriate to the question *“What are the reliable predictors (prognostic factors, variables, tests, scores, and multivariable models), prior to and on admission as well as during the hospital course to predict patient outcome at different follow up time points following each specific disease ?”*, to identify candidate predictors. Screening of articles was completed using DistillerSR software (Evidence Partners, Ottawa, Canada).

Abstract screening (Level I) was performed with the question “Does this study address prognostication in the focus disease (Yes/ No)?”. Pediatric (<16 years) and animal studies were excluded, as were studies evaluating the efficacy of a therapeutic intervention.

Full text screening (Level II) was performed with the following exclusion criteria:

- Sample size less than 35/50/100 patients depending on the prevalence of the disease,
- Studies confined to a mild form the disease,
- Studies focused on a highly selected subgroup (for example penetrating traumatic brain injury, traumatic cardiac arrest, paraneoplastic Guillain-Barré-Syndrome, etc.),
- Studies focused entirely on genetic polymorphism,
- Studies that include multiple disease states and without an adequate sample size and separate analysis of the disease of interest,
- Studies of an intervention,
- Studies with neuroimaging not consistent with contemporary standards used to confirm the diagnosis,
- Studies that did not include one of the selected outcomes for the specific disease,
- Studies of predictors not established as independent with appropriate multivariate analysis,
- Studies of clinical prediction models that did not report model discrimination,
- Studies of laboratory biomarkers were included only if the biomarker was considered clinically relevant, and had been evaluated in more than one published study that met other criteria.

Data extraction and assessment for risk of bias (RoB) and was then performed only for studies that addressed the PICOTS question for each selected candidate predictor AND fulfilled full-text selection criteria. The Quality in Prognostic Studies (QUIPS) RoB instrument was used to evaluate studies of individual prognostic variables,[3] and the Prediction model Risk Of Bias ASsessment Tool (PROBAST) instrument used to evaluate studies of clinical prediction models.[4, 5] In addition to the standard domains of these RoB instruments, studies were evaluated for the risk of bias related to the self-filling prophecy with an additional domain that included three questions- whether a treatment suspension policy was used in the study, whether clinicians were blinded to the predictor and whether the predictor was systematically utilized by clinicians for prognostication during the time period of the study. Following assessment of risk of bias in each domain, an overall risk of bias- *high, moderate or low*- was assigned to each study.

Following data extraction and assessment of risk of bias of individual studies, a GRADE evidence profile with summary of findings table was constructed. Statistical measures of effect size varied across studies and were recorded in narrative form in the summary of findings. Decisions to downgrade the body of evidence for risk were based on review of risk of bias across all individual studies for the PICOTS question. The body of evidence for a specific predictor was downgraded for inconsistency only when the results of studies with approximately equal risk of bias were in conflict, with some studies suggesting the predictor was reliable and other studies showing a lack of statistical significance or a high rate of error. The evidence was downgraded for indirectness when population, predictor, outcome, or time of assessment varied significantly from the specifications of the PICOTS question. Downgrading of the evidence for imprecision was applied when the estimated confidence intervals were thought to be excessive for the clinical question. Publication bias could not be meaningfully evaluated, and the GRADE reasons to upgrade were not thought to be applicable to this body of evidence. Judgments on risk of bias, indirectness, inconsistency and imprecision were inherently subjective and focused on the respective prognostication question.

**Effect size (Predictor accuracy)**

Predictor accuracy is often described using measures such as the odds ratio (OR), which measures the *relative* probability of the outcome when the predictor is present, compared to the probability of the outcome in the absence of the predictor. In the context of neuroprognostication, predictors of good and poor outcomes are considered. However, the absolute probability of the outcome when the predictor is present is also important. For example, an older patient may be statistically more likely to suffer a poor outcome than a younger patient, but a large proportion of older individuals may nevertheless have a good outcome.[6] While surrogates of neurocritical care patients may be interested in factors that increase the probability of poor outcome, the absolute probability of a poor outcome is most likely used to make decisions on escalation or withdrawal of treatment measures. The performance of a clinical prediction model is evaluated using its ability to discriminate binary (good vs poor) outcomes, with measures such as the c-statistic or area under the receiver operating characteristic curve (AUC). Another important measure is model calibration, or the ability to correctly specify the probability of an outcome. Model calibration is typically reported as a goodness of fit, often using the Hosmer-Lemeshow test, or with a calibration curve, slope or intercept.[7]

**Evidence to recommendation criteria**

The GRADE Evidence-to-Recommendation criteria encompassed the overall quality of evidence, balance between desirable and undesirable outcomes, confidence in values and preferences, and resource use. Common principles were established for each disease state by the guideline panel in consultation with the patient or family representative, when considering these criteria.

**Good practice statements**

During literature review, the content experts recognized a lack of direct evidence to support the response to the PICOTS question for the specific disease, often because of insufficient clinical equipoise. Therefore, the panel decided to provide good clinical practice statements. Explicit statement of these clinical practice principles was considered essential to provide context as well as appropriate guidance.

**REFERENCES:**

1. Hemingway, H., Croft, P., Perel, P., et al. Prognosis research strategy (PROGRESS) 1: a framework for researching clinical outcomes*.* BMJ 2013;346:e5595.

2. Riley, R.D., Hayden, J.A., Steyerberg, E.W., et al. Prognosis Research Strategy (PROGRESS) 2: prognostic factor research*.* PLoS Med 2013;10(2):e1001380.

3. Hayden, J.A., van der Windt, D.A., Cartwright, J.L., Cote, P.Bombardier, C. Assessing bias in studies of prognostic factors*.* Ann Intern Med 2013;158(4):280-6.

4. Moons, K.G.M., Wolff, R.F., Riley, R.D., et al. PROBAST: A Tool to Assess Risk of Bias and Applicability of Prediction Model Studies: Explanation and Elaboration*.* Ann Intern Med 2019;170(1):W1-W33.

5. Wolff, R.F., Moons, K.G.M., Riley, R.D., et al. PROBAST: A Tool to Assess the Risk of Bias and Applicability of Prediction Model Studies*.* Ann Intern Med 2019;170(1):51-8.

6. Gattellari, M., Goumas, C., Garden, F.Worthington, J.M. Relative survival after transient ischaemic attack: results from the Program of Research Informing Stroke Management (PRISM) study*.* Stroke 2012;43(1):79-85.

7. Van Calster, B., McLernon, D.J., van Smeden, M., et al. Calibration: the Achilles heel of predictive analytics*.* BMC Med 2019;17(1):230.
