# Supplementary material for: Guidelines for neuroprognostication in adults with traumatic spinal cord injury
Source: Neurocrit Care. 2023 Nov 13;40(2):415–37. doi: 10.1007/s12028-023-01845-8 (PMC10959804; doi:10.1007/s12028-023-01845-8)
Supplement: Supplementary file 2 — (DOCX 14 KB) [file 12028_2023_1845_MOESM2_ESM.docx]

**GRADE Summary: Neurocritical Care Society (NCS)/ German Society for Neuro-Intensive and Emergency Medicine (DGNI) Guidelines for Neuroprognostication: Traumatic Spinal Cord Injury**

**Supplementary Appendix 2: Librarian search string**

Database: All Ovid Medline <1946 - present>

Search Strategy:

--------------------------------------------------------------------------------

1 exp Spinal Cord Injuries/ (45816)

2 Spinal cord injur*.ti,ab. (35004)

3 Spinal cord lacerat*.ti,ab. (3)

4 Spinal cord laceration*.ti,ab. (3)

5 Spinal cord traum*.ti,ab. (892)

6 Spinal cord traum*.ti,ab. (892)

7 (myelopath* adj5 post-traumatic).ti,ab. (36)

8 (myelopath* adj5 traumatic).ti,ab. (148)

9 Spinal cord transect*.ti,ab. (1596)

10 Spinal cord lacerat*.ti,ab. (3)

11 spinal trauma*.ti,ab. (1035)

12 Central Cord Syndrome.tw. (222)

13 exp Central Cord Syndrome/ (89)

14 Spinal Cord Contusion*.ti,ab. (626)

15 *hemiplegia/ or *paraplegia/ or *quadriplegia/ (19055)

16 Quadriplegi*.ti,ab. (3966)

17 Tetraplegi*.ti,ab. (3978)

18 Paraplegi*.ti,ab. (15613)

19 1 or 2 or 3 or 4 or 5 or 6 or 7 or 8 or 9 or 10 or 11 or 12 or 13 or 14 or 15 or 16 or 17 or 18

(83066)

20 exp Analysis of Variance/ [includes Multivariate Analysis] (326771)

21 Multivariate.tw. (293720)

22 treatment outcome/ (885811)

23 outcome.tw. (887975)

24 mortality/ or mo.fs. (563611)

25 "Predictive Value of Tests"/ (188131)

26 Disease Progression/ (146077)

27 prediction.tw. (213326)

28 prognostic*.tw. (268771)

29 prognos*.tw. (536821)

30 exp Quality of Life/ (171899)

31 "Quality of life".tw. (241815)

32 scale.ti,ab. (629667)

33 score.ti,ab. (478944)

34 scoring tool*.tw. (605)

35 disability evaluation/ (44760)

36 Survival/ (4559)

37 mortality/ or mo.fs. or death/ (579364)

38 Time factors/ (1143362)

39 20 or 22 or 23 or 24 or 25 or 27 or 28 or 29 or 30 or 32 or 33 or 34 or 35 or 36 or 38

(4506153)

40 19 and 39 (23774)

41 prognosis.sh. or diagnosed.tw. or cohort:.mp. or predictor:.tw. or death.tw. or exp models,

statistical/ [validated hedge from the Health Information Research Unit, McMaster University]

(2446889)

42 19 and 41 (9563)

43 exp cohort studies/ [includes: follow-up studies/, longitudinal studies/, prospective studies/,

retrospective studies/, controlled before-after studies/, cross-sectional studies/, or historically

controlled study/] (1824723)

44 (Follow-up or longitudinal or prospective or retrospective or before-after or cross-sectional

or controlled).tw. (2597128)

45 (predict* or predictor or prognos* or prognost*).ti. (415631)

46 or/43-45 (3640205)

47 40 or 42 (28179)

48 46 and 47 (11911)

49 animals/ not (animals.mp. and humans/) [mp=title, abstract, original title, name of substance

word, subject heading word, floating sub-heading word, keyword heading word, organism

supplementary concept word, protocol supplementary concept word, rare disease supplementary

concept word, unique identifier, synonyms] (4515460)

50 limit 48 to ("infant (1 to 23 months)" or "preschool child (2 to 5 years)" or "child (6 to 12

years)") (1574)

51 letter.pt. (1015878)

52 case reports.pt. (1923094)

53 49 or 50 or 51 or 52 (7188469)

54 48 not 53 (9009)

***************************
