# Supplementary material for: Guidelines for neuroprognostication in adults with traumatic spinal cord injury
Source: Neurocrit Care. 2023 Nov 13;40(2):415–37. doi: 10.1007/s12028-023-01845-8 (PMC10959804; doi:10.1007/s12028-023-01845-8)
Supplement: Supplementary file 4 — (DOCX 42 KB) [file 12028_2023_1845_MOESM4_ESM.docx]

# **Supplementary Appendix 4. Additional information on predictors of functional outcome**

Based on the a-priori defined methodology for development of these guidelines, scientific publications were deemed insufficient to support recommendations if only one such study supported the evidence. These studies met all other criteria for inclusion, were judged to be of sufficient quality, and are therefore included in the Evidentiary Table of these Guidelines. These studies are incorporated into this Supplementary Appendix to provide additional information for the reader.

**Outcome: Functional outcome (at discharge from rehabilitation or beyond)**

Question: When counseling patients, family members, and/or surrogates of adults with acute traumatic spinal cord injury, should concomitant injuries be considered a reliable predictor of worse functional outcome at discharge from rehabilitation or beyond?

Recommendation: There is insufficient evidence to provide a recommendation.

Rationale: Only one study met all the a-priori criteria to support a recommendation. As a result of multivariate outcome analysis from two combined databases with 376 patients with tSCI, the initial GCS, as an indirect measure of concomitant TBI, did not significantly affect later motor recovery (as per FIM motor score) [1]. High-energy injury mechanisms directly affect spine-related injuries and thus may lead to more severe fracture types or dislocation of the spinal column and therefore more severe tSCI. Similarly, other organ systems are significantly at risk leading to higher degrees of multiple trauma types. The risk of attrition bias is inherent in the study of patients with severe trauma / multiple trauma.

Question: When counseling patients, family members, and/or surrogates of adults with acute traumatic spinal cord injury, should pathological findings on MRI be considered a reliable predictor of worse functional outcome at discharge from rehabilitation or beyond?

Recommendation: There is insufficient evidence to provide a recommendation.

Rationale: Only one study met all the a-priori criteria to support a recommendation. Potential bias was present in the QUIPS domains of study attrition, outcome measurement, statistical analysis and reporting. This included acute clinical and imaging factors in for the derivation of a prognostic model [2]. Signal characteristics consistent with spinal cord edema or hemorrhage led to stepwise prediction of worse functional outcome in a linear model predicting FIM motor scores at 1 year. The study was eliminated from further analysis in the prediction model section because of lack of external validation. Although improvement in prognostication by clinico-radiological biomarkers appears inherently logical, one must consider that the same findings may ultimately trigger additional treatment such as early spinal canal decompression which will in turn alter neurological outcome. Unless spinal cord transection is clearly demonstrated, pathological findings such as spinal canal compromise or intramedullary signal changes are inconsistent in predicting future functional improvement.

**Outcome: AIS Improvement (conversion) (at discharge from rehabilitation or beyond)**

Question: When counseling patients, family members, and/or surrogates of adults with acute traumatic spinal cord injury, should comorbidities be considered a reliable predictor of AIS conversion at discharge from rehabilitation or beyond?

Recommendation: There is insufficient evidence to provide a recommendation.

Rationale: Only one study met all the a-priori criteria to support a recommendation. Potential bias was present in the QUIPS domains of study participation, study confounding and self-fulfilling prophecy. The available evidence for the effect of comorbidities on AIS conversion following tSCI is scarce. In a monocentric study of 57 patients that enrolled only patients operated for cervical spine fractures, comorbidities as measured by the CCI had a significantly negative effect in univariate analysis with more comorbidities in the group without functional improvement. Similar associations were seen with higher age and deferred surgical timing (>12hrs). Multivariate analyses, however, revealed no independent predictive value of the CCI for future AIS conversion [3]. Given only one study (with small sample size) met all requirements, but only in univariate and not multivariate analysis, the conclusion was drawn not to consider CCI as a reliable predictor.

**Outcome: Independent ambulation (at discharge from rehabilitation or beyond)**

Question: When counseling patients, family members, and/or surrogates of adults with acute traumatic spinal cord injury, should *age OR neurologic level of injury OR severity of injury as measured by the AIS* at time of injury be considered a reliable predictor of future independent ambulation at discharge from rehabilitation or beyond?

Recommendation: There is insufficient evidence to provide a recommendation.

Rationale: One study met inclusion criteria and demonstrated risk of bias in the QUIPS domains of study participation and attrition, confounding, and self-fulfilling prophecy. Although older age seems to have an impact on the natural course of recovery from neurological injuries such as tSCI, age thresholds (e.g., >65 yrs or >50 yrs) vary across studies and are arbitrary. In a study of 400 patients of patients with thoracic tSCI, the odds ratios for walking at 1-year post injury were not significantly affected by age (OR 1.8; 0.8-4.0), but were impacted by the initial AIS grade and whether the NLI was in the upper or lower thoracic spinal cord [4]. While in tetraplegic patients motor improvement is typically seen in the upper extremities, improvement in lower extremity strength also occurs. In this study the odds for independent ambulation at 1 year increased for thoracic tSCI below T9 as compared to higher thoracic injuries. Multivariate analysis revealed an odds ratio of 2.4 (1.2-4.9). Only 5.4% of patients with complete (AIS A) thoracic or thoraco-lumbar injuries recovered to independent ambulation. In comparison the odds for independent ambulation at 1 year in patients with AIS B-D injuries increased to an OR 6.0 (2.7- 13.5), OR 30.4 (12.5-74.0), and OR 72.6 (18.5-285.0), respectively [4]. Thus, in multivariate analysis all three factors were independent predictors of future ambulatory potential. This study may serve as a positive example to guide future study and model development to help support a recommendation.

REFERENCES

[1] J.R. Wilson, A.M. Davis, A.V. Kulkarni, A. Kiss, R.F. Frankowski, R.G. Grossman, M.G. Fehlings, Defining age-related differences in outcome after traumatic spinal cord injury: analysis of a combined, multicenter dataset, The Spine Journal. 14 (2014) 1192–1198. https://doi.org/10.1016/j.spinee.2013.08.005.

[2] J.R. Wilson, R.G. Grossman, R.F. Frankowski, A. Kiss, A.M. Davis, A.V. Kulkarni, J.S. Harrop, B. Aarabi, A. Vaccaro, C.H. Tator, M. Dvorak, C.I. Shaffrey, S. Harkema, J.D. Guest, M.G. Fehlings, A Clinical Prediction Model for Long-Term Functional Outcome after Traumatic Spinal Cord Injury Based on Acute Clinical and Imaging Factors, Journal of Neurotrauma. 29 (2012) 2263–2271. https://doi.org/10.1089/neu.2012.2417.

[3] M. Dobran, M. Iacoangeli, N. Nocchi, A. Di Rienzo, L.G. di Somma, D. Nasi, R. Colasanti, M. Al-Fay, M. Scerrati, Surgical treatment of cervical spine trauma: Our experience and results, Asian J Neurosurg. 10 (2015) 207. https://doi.org/10.4103/1793-5482.161192.

[4] B.A. Lee, B.E. Leiby, R.J. Marino, Neurological and functional recovery after thoracic spinal cord injury, J Spinal Cord Med. 39 (2016) 67–76. https://doi.org/10.1179/2045772314Y.0000000280.
