# Supplementary material for: Guidelines for neuroprognostication in adults with traumatic spinal cord injury
Source: Neurocrit Care. 2023 Nov 13;40(2):415–37. doi: 10.1007/s12028-023-01845-8 (PMC10959804; doi:10.1007/s12028-023-01845-8)
Supplement: Supplementary file 5 — (DOCX 14 KB) [file 12028_2023_1845_MOESM5_ESM.docx]

| Supplementary Table 1. Charlson comorbidity index (CCI sum score) | | |
| --- | --- | --- |
| **Comorbidity** | **Score** |  |
| Prior myocardial infarction | 1 |  |
| Congestive heart failure | 1 |  |
| Peripheral vascular disease | 1 |  |
| Cerebrovascular disease | 1 |  |
| Dementia | 1 |  |
| Chronic pulmonary disease | 1 |  |
| Rheumatologic disease | 1 |  |
| Peptic ulcer disease | 1 |  |
| Mild liver disease | 1 |  |
| Diabetes | 1 |  |
| Cerebrovascular (hemiplegia) event | 2 |  |
| Moderate-to-severe renal disease | 2 |  |
| Diabetes with chronic complications | 2 |  |
| Cancer without metastases | 2 |  |
| Leukemia | 2 |  |
| Lymphoma | 2 |  |
| Moderate or severe liver disease | 3 |  |
| Metastatic solid tumor | 6 |  |
| Acquired immune-deficiency syndrome (AIDS) | 6 |  |
| *Charlson ME, Pompei P, Ales KL, MacKenzie CR. A new method of classifying prognostic comorbidity in longitudinal studies: development and validation. J Chronic Dis. 1987;40:373–383.* | |  |
