# Supplementary material for: Guidelines for neuroprognostication in adults with traumatic spinal cord injury
Source: Neurocrit Care. 2023 Nov 13;40(2):415–37. doi: 10.1007/s12028-023-01845-8 (PMC10959804; doi:10.1007/s12028-023-01845-8)
Supplement: Supplementary file 6 — (DOCX 14 KB) [file 12028_2023_1845_MOESM6_ESM.docx]

| Supplementary Table 2. American Spinal Injury Association (ASIA) Impairment Scale, AIS | |
| --- | --- |
| A | Complete: No sensory or motor function is preserved in sacral segments S4-S5 |
| B | Incomplete: Sensory, but not motor, function is preserved below the neurologic level and extends through sacral segments S4-S5 |
| C | Incomplete: Motor function is preserved below the neurologic level, and most key muscles below the neurologic level have a muscle grade of less than 3 |
| D | Incomplete: Motor function is preserved below the neurologic level, and most key muscles below the neurologic level have a muscle grade that is greater than or equal to 3 |
| E | Normal: Sensory and motor functions are normal |
| *https://asia-spinalinjury.org/international-standards-neurological-classification-sci-isncsci-worksheet/* | |
