# Supplementary material for: Guidelines for neuroprognostication in adults with traumatic spinal cord injury
Source: Neurocrit Care. 2023 Nov 13;40(2):415–37. doi: 10.1007/s12028-023-01845-8 (PMC10959804; doi:10.1007/s12028-023-01845-8)
Supplement: Supplementary file 7 — (DOCX 14 KB) [file 12028_2023_1845_MOESM7_ESM.docx]

| Supplementary Table 3. Functional Independence Measure (FIM) |
| --- |
|  |
| **Self-Care** |
| A. Eating |
| B. Grooming |
| C. Bathing |
| D. Dressing - Upper Body |
| E. Dressing - Lower Body |
| F. Toileting |
| **Sphincter Control** |
| G. Bladder Management |
| H. Bowel Management |
| **Transfers** |
| I. Bed, Chair, Wheelchair |
| J. Toilet |
| K. Tub, Shower |
| **Locomotion** |
| L. Walk/Wheelchair |
| M. Stairs |
| *Motor Subtotal Score* |
| **Communication** |
| N. Comprehension |
| O. Expression |
| **Social Cognition** |
| P. Social Interaction |
| Q. Problem Solving |
| R. Memory |
| *Cognitive Subtotal Score* |
| **TOTAL FIM Score** |

| **L**  **E**  **V**  **E**  **L**  **S** | **Independent**  7 Complete Independence (Timely, Safely)  6 Modified Independence (Device) | **NO HELPER** |
| --- | --- | --- |
|  | **Modified Dependence**  5 Supervision (Subject = 100%+)  4 Minimal Assist (Subject = 75%+)  3 Moderate Assist (Subject = 50%+)  **Complete Dependence**  2 Maximal Assist (Subject = 25%+)  1 Total Assist (Subject = less than 25%) | **HELPER** |

Heinemann AW et al. Arch Phys Med Rehabil. 1993. 74: 566-573.
