# Supplementary material for: Guidelines for neuroprognostication in adults with traumatic spinal cord injury
Source: Neurocrit Care. 2023 Nov 13;40(2):415–37. doi: 10.1007/s12028-023-01845-8 (PMC10959804; doi:10.1007/s12028-023-01845-8)
Supplement: Supplementary file 8 — (DOCX 23 KB) [file 12028_2023_1845_MOESM8_ESM.docx]

| Supplementary Table 4:  Spinal Cord Independence Measure Version III (SCIM-III ) |
| --- |

SCIM III (total score 0-100)

*Self-Care (subtotal 0-20)*

**1: Feeding (cutting, opening containers, pouring, bringing food to mouth, holding cup with fluid):**

0: Needs parenteral, gastrostomy, or fully assisted oral feeding

1: Needs partial assistance for eating and/or drinking, or for wearing adaptive devices

2: Eats independently; needs adaptive devices or assistance only for cutting food and/or pouring and/or opening containers

3: Eats and drinks independently; does not require assistance or adaptive devices

**2: Bathing (soaping, washing, drying body and head, manipulating water tap)**

*A: upper body:*

0: Requires total assistance

1: Requires partial assistance

2: Washes independently with adaptive devices or in a specific setting (e.g., bars, chair)

3: Washes independently; does not require adaptive devices or specific setting (not customary for healthy people) (adss)

*B: lower body:*

0: Requires total assistance

1: Requires partial assistance

2: Washes independently with adaptive devices or in a specific setting (adss)

3: Washes independently; does not require adaptive devices (adss) or specific setting

**3: Dressing (clothes, shoes, permanent orthoses: dressing, wearing, undressing).**

*A: upper body:*

0: Requires total assistance

1: Requires partial assistance with clothes without buttons, zippers or laces (cwobzl)

2: Independent with cwobzl; requires adaptive devices and/or specific settings (adss)

3: Independent with cwobzl; does not require adss; needs assistance or adss only for bzl

4: Dresses (any cloth) independently; does not require adaptive devices or specific setting

*B: lower body:*

0: Requires total assistance

1: Requires partial assistance with clothes without buttons, zippers or laces (cwobzl)

2: Independent with cwobzl; requires adaptive devices and/or specific settings (adss)

3: Independent with cwobzl without adss; needs assistance or adss only for bzl

4: Dresses (any cloth) independently; does not require adaptive devices or specific setting

**4: Grooming (washing hands and face, brushing teeth, combing hair, shaving, applying makeup): ***

0: Requires total assistance

1: Requires partial assistance

2: Grooms independently with adaptive devices

3: Grooms independently without adaptive devices

*Respiration and Sphincter Management (subtotal 0-40)*

**5: Respiration:**

0: Requires tracheal tube (TT) and permanent or intermittent assisted ventilation (IAV)

2: Breathes independently with TT; requires oxygen, much assistance in coughing or TT management

4: Breathes independently with TT; requires little assistance in coughing or TT managemen

6: Breathes independently without TT; requires oxygen, much assistance in coughing, a mask (e.g., peep) or IAV (bipap)

8: Breathes independently without TT; requires little assistance or stimulation for coughing

10: Breathes independently without assistance or device

**6: Sphincter Management - Bladder:**

0: Indwelling catheter

3: Residual urine volume (RUV) > 100cc; no regular catheterization or assisted intermittent catheterization

6: RUV < 100cc or intermittent self-catheterization; needs assistance for applying drainage instrument

9: Intermittent self-catheterization; uses external drainage instrument; does not need assistance for applying

11: Intermittent self-catheterization; continent between catheterizations; does not use external drainage instrument

13: RUV <100cc; needs only external urine drainage; no assistance is required for drainage

15: RUV <100cc; continent; does not use external drainage instrument

**7: Sphincter Management - Bowel:**

0: Irregular timing or very low frequency (less than once in 3 days) of bowel movements

5: Regular timing, but requires assistance (e.g., for applying suppository); rare accidents (less than twice a month)

8: Regular bowel movements, without assistance; rare accidents (less than twice a month)

10: Regular bowel movements, without assistance; no accidents

**8: Use of Toilet (perineal hygiene, adjustment of clothes before/after, use of napkins or diapers):**

0: Requires total assistance

1: Requires partial assistance; does not clean self

2: Requires partial assistance; cleans self independently

4: Uses toilet independently in all tasks but needs adaptive devices or special setting (e.g., bars)

5: Uses toilet independently; does not require adaptive devices or special setting

*Mobility (room and toilet) (subtotal 0-10)*

**9: Mobility in Bed and Action to Prevent Pressure Sores:**

0: Needs assistance in all activities: turning upper body in bed, turning lower body in bed,sitting up in bed, doing push-ups in wheelchair, with or without adaptive devices, but not with electric aids

2: Performs one of the activities without assistance

4: Performs two or three of the activities without assistance

6: Performs all the bed mobility and pressure release activities independently

**10: Transfers: bed-wheelchair (locking wheelchair, lifting footrests, removing and adjusting arm rests, transferring, lifting feet):**

0: Requires total assistance

1: Needs partial assistance and/or supervision, and/or adaptive devices (e.g., sliding board)

2: Independent (or does not require wheelchair)

**11: Transfers: wheelchair-toilet-tub (if uses toilet wheelchair: transfers to and from; if uses regular wheelchair: locking** wheelchair, lifting footrests, removing and adjusting armrests, transferring, lifting feet):

0: Requires total assistance

1: Needs partial assistance and/or supervision, and/or adaptive devices (e.g., grab-bars)

2: Independent (or does not require wheelchair)

*Mobility (indoors and outdoors, on even surface) (subtotal 0-30)*

**12: Mobility Indoors:**

0: Requires total assistance

1: Needs electric wheelchair or partial assistance to operate manual wheelchair

2: Moves independently in manual wheelchair

3: Requires supervision while walking (with or without devices)

4: Walks with a walking frame or crutches (swing)

5: Walks with crutches or two canes (reciprocal walking)

6: Walks with one cane

7: Needs leg orthosis only

8: Walks without walking aids

**13: Mobility for Moderate Distances (10-100 meters):**

0: Requires total assistance

1: Needs electric wheelchair or partial assistance to operate manual wheelchair

2: Moves independently in manual wheelchair

3: Requires supervision while walking (with or without devices)

4: Walks with a walking frame or crutches (swing)

5: Walks with crutches or two canes (reciprocal walking)

6: Walks with one cane

7: Needs leg orthosis only

8: Walks without walking aids

**14:Mobility Outdoors (more than 100 meters):**

0: Requires total assistance

1: Needs electric wheelchair or partial assistance to operate manual wheelchair

2: Moves independently in manual wheelchair

3: Requires supervision while walking (with or without devices)

4: Walks with a walking frame or crutches (swing)

5: Walks with crutches or two canes (reciprocal waking)

6: Walks with one cane

7: Needs leg orthosis only

8: Walks without walking aids

**15: Stair Management:**

0: Unable to ascend or descend stairs

1: Ascends and descends at least 3 steps with support or supervision of another person

2: Ascends and descends at least 3 steps with support of handrail and/or crutch or cane

3: Ascends and descends at least 3 steps without any support or supervision

**16: Transfers: wheelchair-car (approaching car, locking wheelchair, removing arm- and footrests, transferring to and from car,** bring wheelchair into and out of car):

0: Requires total assistance

1: Needs partial assistance and/or supervision and/or adaptive devices

2: Transfers independent; does not require adaptive devices (or does not require wheelchair)

**17: Transfers: ground-wheelchair:**

0: Requires assistance

1: Transfers independent with or without adaptive devices (or does not require wheelchair)

*Catz A, Itzkovich M. JRRD. 2007;44(1):65-68.*
